# UVB radiation suppresses Dicer expression through β-catenin

**DOI:** 10.1242/jcs.261978

**Published:** 2024-11-26

**Authors:** Zackie Aktary, Valérie Petit, Irina Berlin, Jeremy Raymond, Frederique Berger, Nisamanee Charoenchon, Evelyne Sage, Juliette Bertrand, Lionel Larue

**Affiliations:** ^1^ Institut Curie, PSL Research University, INSERM U1021, Normal and Pathological Development of Melanocytes, 91405 Orsay, France; ^2^ Université Paris-Saclay, Univ Paris-Saclay, CNRS UMR 3347, 91405 Orsay, France; ^3^ Ludwig Institute for Cancer Research, Nuffield Department of Medicine, University of Oxford, 0X3 7DQ, Headington, Oxford, UK; ^4^ Department of Biostatistics, Institut Curie, 92210 Saint-Cloud, France

**Keywords:** β-catenin, Dicer, UVB, Transcription, Melanocytes, Melanoma

## Abstract

Ultraviolet (UV) rays prompt a natural response in epidermal cells, particularly within melanocytes. The changes in gene expression and related signaling pathways in melanocytes following exposure to UV radiation are still not entirely understood. Our findings reveal that UVB irradiation suppresses the expression of Dicer (also known as Dicer1). This repression is intricately linked to the activation of the phosphoinositide 3-kinase (PI3K), ribosomal S6 kinase (RSK) and Wnt–β-catenin signaling pathways, and is directly associated with transcriptional repression by β-catenin (also known as CTNNB1). Notably, we have identified specific binding sites for the TCF/LEF–β-catenin complex in the Dicer promoter. Collectively, these results emphasize the significance of the UV-induced pathway involving the TCF/LEF–β-catenin complex, which impacts Dicer expression. UV radiation also reduced the levels of specific microRNAs known to be important in the biology of melanocytes. This pathway holds potential importance in governing melanocyte physiology.

## Teaser

**Summary:** The skin is exposed to ultraviolet radiation. Here, we show that UVB induces Wnt–β-catenin signaling that leads to downregulation of Dicer and a reduction in levels of microRNAs involved in melanocyte biology.

## INTRODUCTION

Gene regulation is a central process in the life of cells, as well as in communication between cells and their (micro-)environment. Among the key players in this intricate network is Dicer (also known as Dicer1), an enzyme that plays a pivotal role in governing the production of small RNA molecules that are crucial for modulating gene expression. It has been demonstrated that Dicer activity is subject to regulation by various factors, including reactive oxygen species (ROS), hypoxia, serum, interferons and phorbol esters (Asada et al., 2008; van den Beucken et al., 2014; Wiesen and Tomasi, 2009).

Dicer is a multifaceted enzyme belonging to the ribonuclease III family and acts as a conductor of gene expression by finely adjusting the levels of small RNA molecules, including microRNAs (miRNAs) and small interfering RNAs (siRNAs). These small RNA molecules, typically composed of 20–30 nucleotides, play a critical role in post-transcriptional gene regulation, dictating the fate of target mRNAs by either promoting their degradation or inhibiting their translation (Bartel, 2018). Under conditions of oxidative stress, there is a surge in ROS levels, triggering a cascade of cellular responses. Among these responses is the downregulation of Dicer expression and activity, resulting in altered miRNA processing and subsequent changes in gene expression patterns (Chakraborty et al., 2017; Grishok et al., 2001; Kozomara and Griffiths-Jones, 2014; Wiesen and Tomasi, 2009). Phorbol esters – which are capable of mimicking the effects of diacylglycerol, a potent activator of protein kinase C (PKC) – can induce the phosphorylation of Dicer, leading to enhanced miRNA processing and subsequent alterations in gene expression profiles (Shin et al., 2009). However, they can also repress the expression of Dicer (Wiesen and Tomasi, 2009). Additionally, the presence of double-stranded RNA and interferon-α proteins has been found to suppress Dicer production, while interferon-γ (also known as IFNG) induces it. Serum withdrawal and hypoxia downregulate Dicer, increasing susceptibility to apoptosis (Asada et al., 2008; van den Beucken et al., 2014; Wiesen and Tomasi, 2009).

In the context of skin biology, Dicer has been shown to play a pivotal role in essential aspects of hair follicle and epidermal morphogenesis and maintenance (Andl et al., 2006; Vishlaghi and Lisse, 2020). In melanocytes, Dicer has been associated with apoptosis and disruption of proper melanocyte migration within the growing hair follicle, ultimately leading to the depletion of the pool of melanocyte stem cells (Bertrand et al., 2024; Levy et al., 2010). Cells of the skin, including melanocytes, keratinocytes and dermal fibroblasts, are continuously exposed to the potentially harmful effects of ultraviolet (UV) radiation from sunlight, which can lead to DNA damage, oxidative stress, inflammation and changes in gene expression. To mitigate these risks, this organ has developed a variety of defense mechanisms. Different cell types employ both general and specific strategies for defense. General protective measures include processes such as cell cycle inhibition, replication suppression, DNA repair and apoptosis. Specifically, keratinocytes produce α-melanocyte-stimulating hormone, which signals to melanocytes to increase melanin production, whereas fibroblasts generate more metalloproteinases, which break down collagen and elastin fibers – essential components of the extracellular matrix. Dysfunctions in these protective mechanisms can result in a range of pathophysiological conditions, including premature skin aging and manifestations of sun damage, such as wrinkles, leathery skin, liver spots, actinic keratosis, solar elastosis, loss of pigmentation and the development of melanoma, a highly aggressive and increasingly prevalent form of skin cancer. UV exposure has been definitively linked to the emergence of skin conditions, including melanoma (Gorman et al., 2019), with epidemiological studies suggesting that a substantial proportion of melanoma cases, ranging from 65% to 90%, can be attributed to UV exposure (Glanz et al., 2007). Within the solar spectrum, UVB light (ranging from 290 nm to 320 nm) has been identified as a pivotal factor in the induction of melanoma (De Fabo et al., 2004; Gaffal et al., 2011; Mitchell and Fernandez, 2011). Furthermore, the involvement of Dicer in the stress response and its regulation under various cellular and environmental conditions underscore the importance of understanding the context-specific modulation of Dicer expression. However, the precise mechanism governing the control of Dicer expression remains elusive, as only a limited number of upstream factors that modulate its activity, including ROS, hypoxia, serum, interferons and phorbol esters, have been identified (Asada et al., 2008; Levy et al., 2010; Martello et al., 2010; Su et al., 2010; Wiesen and Tomasi, 2009). Given the critical role of melanocytes in shielding the skin against the harmful effects of UV radiation, this study aimed to investigate the regulation of Dicer gene expression in response to UV stress within the melanocyte lineage.

## RESULTS AND DISCUSSION

### UVB represses Dicer at the transcriptional level

**Fig. 1.**
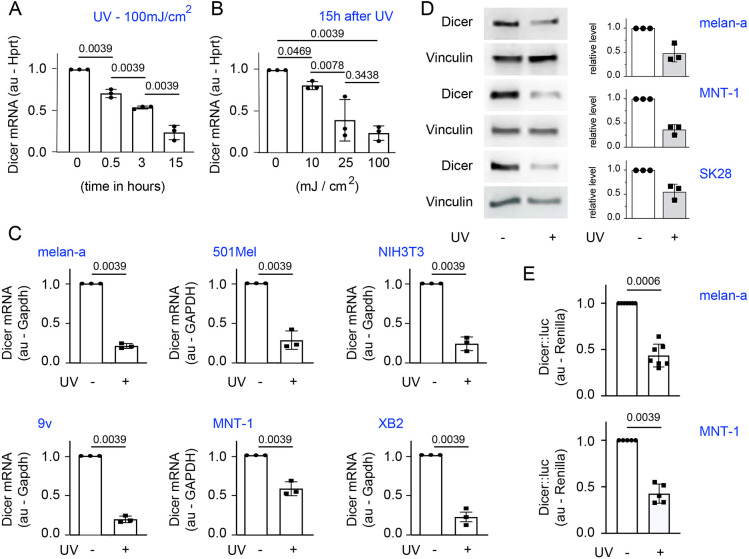
**UVB irradiation represses Dicer expression in various cell types.** (A–C) Dicer transcript levels were assessed using RT-qPCR following UV irradiation or without UV exposure. (A,B) RNA was extracted from melan-a cells 0–15 h after UVB irradiation at 100 mJ/cm^2^ (A), or 15 h after UVB irradiation ranging from 0 mJ/cm^2^ to 100 mJ/cm^2^ (B). Dicer transcript readouts were normalized to *Hprt*. (C) RNA was collected from melan-a and 9v mouse melanocytes, NIH3T3 mouse fibroblasts, and XB2 mouse keratinocytes at 15 h after UVB irradiation at 100 mJ/cm^2^ (+) or without irradiation (−). Similar experiments were performed with 501Mel and MNT-1 human melanoma cells with or without UVB irradiation at 25 mJ/cm^2^. The Dicer transcript readouts were normalized to *Gapdh* for mouse cell lines and to *GAPDH* for human cell lines. (D) Dicer protein levels were analyzed by western blotting of samples from melan-a mouse melanocytes and from MNT-1 and SK28 human melanoma cells 27 h after UVB irradiation (100 mJ/cm^2^ for all cells) or without UVB exposure. Left: a representative western blot is presented for each cell line, with vinculin used as the loading control. Right: quantification of relative Dicer protein levels normalized to the non-irradiated controls. (E) Dicer::luciferase (Dicer::luc) transcriptional activity was measured 15 h after UVB irradiation (+) in melan-a cells (100 mJ/cm^2^) or in MNT-1 cells (25 mJ/cm^2^). Cells without irradiation (−) were used as a control. Dicer::luciferase activity was normalized to Renilla luciferase and is presented relative to the activity in control cells. Data in A–E are presented as mean±s.d. The presented data correspond to experiments that were conducted biologically at least three times with three independent technical replicates for each. Statistical analyses in A–C and D were performed using a Wilcoxon matched-pairs signed-rank test. The *P*-values are given in the figure. Au, arbitrary units.

Given that UVB radiation primarily affects epidermal cells, we determined the effect of UVB radiation on Dicer expression in melanocytes. Dicer mRNA levels in melan-a mouse melanocyte cells were found to decrease in a dose- and time-dependent manner when the cells were exposed to UVB radiation, as determined by reverse transcription-quantitative PCR (RT-qPCR) (Fig. 1A–C). Further experiments were performed using 25 mJ/cm^2^ or 100 mJ/cm^2^ UVB, and the level of Dicer was determined 15 h after irradiation. The level of Dicer mRNA was found to decrease after UVB exposure across various cell lines, encompassing mouse melanocytes (melan-a and 9v), fibroblasts (NIH3T3) and keratinocytes (XB2), as well as the human melanoma cell lines 501Mel and MNT-1 (Fig. 1C; Fig. S1). As expected, the level of Dicer protein was decreased across transformed and non-transformed cell lines following UVB exposure (Fig. 1D). These findings indicate that the reduction in Dicer expression following UVB exposure is not specific to the melanocyte lineage and is species independent. This decline in Dicer protein and mRNA levels after melanocyte exposure to UVB suggested that Dicer transcription is affected in response to UVB irradiation. To further explore this mechanism, we performed reporter assays in melan-a and MNT-1 cells with a luciferase reporter construct driven by the Dicer promoter. The results indicated that Dicer promoter activity was halved following UVB irradiation compared to the activity in mock-treated cells, providing evidence that the response of Dicer to UVB irradiation occurs, at least partially, at the transcriptional level (Fig. 1E). These findings indicate that the regulation of Dicer transcription following UVB exposure is species independent. Interestingly, UV radiation, potentially UVC, has been shown to suppress Dicer mRNA expression in specific cells such as mouse preadipocytes (Mori et al., 2012). However, the underlying mechanism behind this suppression remains unreported.

### Dicer is regulated by UV through the GSK3β–β-catenin pathway

**Fig. 2.**
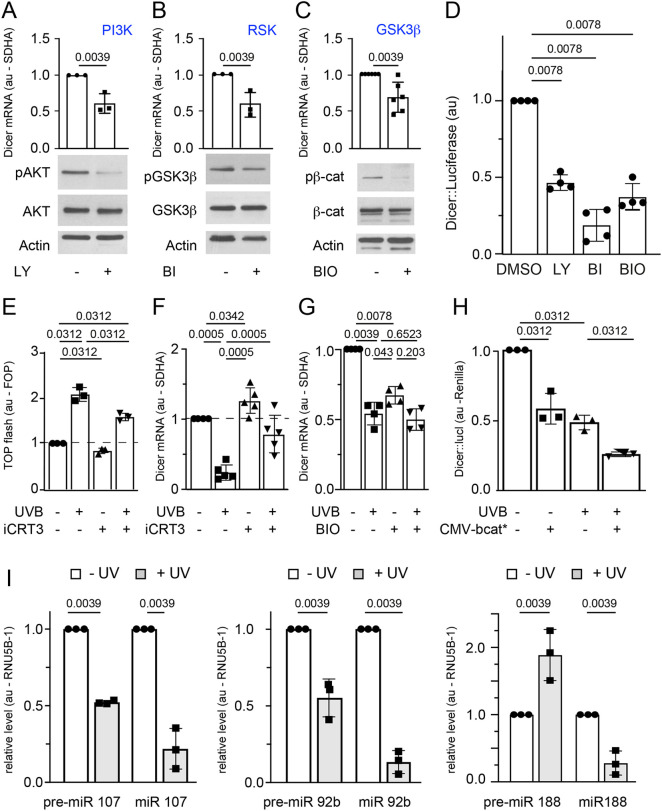
**Regulation of Dicer via PI3K, RSK and GSK3β pathways.** (A–G) The effect of various signaling pathways on the expression of Dicer in MNT-1 cells was assessed using different chemical inhibitors. (A–C) The PI3K inhibitor LY294002 (LY) was used at 50 μM (A), the RSK inhibitor BI-D1870 (BI) was used at 20 μM (B) and the GSK3β inhibitor BIO was used at 5 μM (C). All chemical inhibitor treatments lasted 6 h. DMSO was used as a vehicle control. The effectiveness of each inhibitor was validated by assessing the phosphorylation levels of downstream signaling proteins in treated (+) and untreated (−) cells using western blotting. pAKT, AKT proteins phosphorylated at S473; pGSK3β, GSK3β phosphorylated at S9; pβ-cat, β-catenin phosphorylated at S33, S37 and T41. Actin is shown as a loading control. The blots shown are representative of at least three biologically independent experiments. The Dicer mRNA levels, as assessed using RT-qPCR, were normalized to *SDHA* and are expressed relative to levels in control cells. (D) Effect of LY294002, BIO and BI-D1870 on Dicer expression, as assessed using a Dicer::luciferase reporter construct (Levy et al., 2010), following a 24 h treatment with each inhibitor. Inhibitor concentrations were as in A–C. DMSO was used as a vehicle control. Dicer::luciferase activity was normalized to Renilla luciferase and is presented relative to the activity in DMSO-treated cells. (E) The efficacy of iCRT3, the TCF/LEF–β-catenin inhibitor, was validated by assessing TOP-flash activity (Korinek et al., 1997) 6 h after treatment with 10 μM iCRT3 or DMSO as a control in the presence or absence of UVB (100 mJ/cm^2^). FOP, FOP-flash activity was used as a normalizing control. (F,G) Effect of 6 h treatment of UVB (100 mJ/cm^2^) in conjunction with either 10 μM iCRT3 (F) or 5 μM BIO (G) on Dicer mRNA levels as assessed by RT-qPCR. DMSO was used as a vehicle control. The Dicer mRNA levels were normalized to *SDHA* and are expressed relative to levels in untreated control cells. (H) Effect of 6 h UVB (100 mJ/cm^2^) treatment and/or β-catenin expression (CMV-bcat*) on Dicer::luciferase (Dicer::luc) transcriptional activity, as assessed in MNT-1 cells. Dicer::luciferase activity was normalized to Renilla luciferase and is presented relative to the activity in untreated control cells. (I) Effect of 27 h UVB (100 mJ/cm^2^) on the levels of selected miRNAs and pre-miRNAs in MNT-1 cells, as assessed using RT-qPCR. Levels were normalized to RNU5B-1 and are expressed relative to levels in untreated cells. Data in A–I are presented as mean±s.d. The presented quantitative data correspond to experiments that were conducted biologically at least three times with three independent technical replicates for each. Statistical analysis was performed using a Wilcoxon matched-pairs signed-rank test. The *P*-values are given in the figure. Au, arbitrary units.

To determine which pathways regulate Dicer within the melanocyte lineage, we treated MNT-1 cells with pharmacological inhibitors targeting various signaling pathways – including the mitogen-activated protein kinase (MAPK), phosphoinositide 3-kinase (PI3K), ATM, PKC and ribosomal S6 kinase (RSK) pathways – before assessing the level of endogenous Dicer mRNA. We assessed the effectiveness of each inhibitor by evaluating their impact on their known targets by western blot analysis. Inhibitors of ATM (KU-55933) and PKC (Enzastaurin) did not affect Dicer mRNA levels (Fig. S1J,K). Conversely, inhibitors of PI3Ks (LY294002), RSKs (BI-D1870) and MAPK kinases (MEKs; U0126) reduced Dicer mRNA levels to 60%, 50% and 85% of those in untreated controls, respectively (Fig. 2A,B; Fig. S1I). As the reduction of Dicer mRNA levels was low in the presence of MEK inhibitor, the impact of this pathway would be minimal compared with that of the PI3K and RSK signaling pathways. In addition, our results indicate that PI3K and RSK proteins play a more prominent role in regulating Dicer mRNA expression. Interestingly, both the PI3K and RSK pathways indirectly induce inhibitory phosphorylation of GSK3β (GSK3B) at serine 9 (De Mesquita et al., 2001; Fang et al., 2000). Treating MNT-1 cells with a direct GSK3β inhibitor, BIO, reduced Dicer mRNA levels to 70% of those in untreated controls (Fig. 2C), demonstrating that GSK3β also regulates Dicer mRNA levels. Inhibition of PI3Ks (using LY294002), RSKs (using BI-D1870) and GSK3β (using BIO) also decreased the expression of a Dicer luciferase reporter construct (Fig. 2D).

The best documented signaling function of GSK3β kinase is its phosphorylation of β-catenin (also known as CTNNB1) at serine 33, serine 37 and threonine 41, leading to its subsequent inactivation (Peifer et al., 1994). Active β-catenin, in conjunction with transcription factors, particularly those from the TCF/LEF family, regulates transcription. Furthermore, we have previously demonstrated that UVB results in the nuclear accumulation of β-catenin and its subsequent transcriptional activity in mouse melanocytes (Bertrand et al., 2017). To determine whether GSK3β-mediated regulation of Dicer mRNA expression is modulated by β-catenin, MNT-1 cells were treated with iCRT3, which specifically inhibits the TCF/LEF–β-catenin interaction. The activity of iCRT3 was validated using a TOP-flash reporter assay (Fig. 2E) before we analyzed the endogenous expression of Dicer mRNA. The iCRT3 inhibitor increased Dicer mRNA levels to 130% of those in the untreated control (Fig. 2F). Importantly, iCRT3 treatment abrogated the decreased levels of Dicer mRNA observed after UVB exposure (Fig. 2F). In addition, although the GSK3β inhibitor BIO decreased Dicer mRNA levels, there was no additive effect of BIO treatment and UVB exposure in decreasing Dicer mRNA levels (Fig. 2G), suggesting that there is no major cooperation between UVB and BIO under these specific experimental conditions. Furthermore, β-catenin expression and/or UVB exposure reduced the level of Dicer transcription activity (Fig. 2H). Hence, both GSK3β and β-catenin proteins participate in the regulation of Dicer mRNA expression. This hypothesis is further supported by previously published data indicating that β-catenin is capable of binding to the promoter region of Dicer (Bottomly et al., 2010). Although our results are the first demonstration of β-catenin regulating Dicer in melanocytes, the relationship between β-catenin and Dicer has previously been shown in the HEYA8 ovarian cancer cell line by a classical functional analysis involving both overexpression and suppression of the level of β-catenin (To et al., 2017). However, the precise mechanism through which β-catenin represses Dicer remained unexplored at the time (To et al., 2017). With β-catenin being a prominent downstream effector of the Wnt pathway, it is possible that activation of this pathway could potentially suppress Dicer. Although this possibility is intriguing, it has not yet been documented in the literature. Furthermore, there may be a potential negative feedback loop from Dicer to β-catenin, as overexpression of Dicer has been shown to decrease β-catenin expression and Wnt signaling, as observed in studies involving *Xenopus* and mice (Tagami et al., 2021; Wu et al., 2019). Our results also suggest that UVB-mediated Dicer repression is not cell-type specific, since we observed similar results in melanocyte, keratinocyte and fibroblast cell lines.

We chose to look at the expression of three miRNAs (miR92b, miR107 and miR188) that have previously been shown to be positively regulated by Dicer in melanocytes (Levy et al., 2010; Bertrand et al., 2024). We hypothesized that UVB exposure, which decreases Dicer expression, would decrease the levels of these miRNAs, in comparison with the associated pre-miRNA gene expression. Indeed, we observed that in MNT-1 cells, UVB exposure decreased the expression of mature miR92b to 13% of expression levels in the non-irradiated controls (Fig. 2I), whereas the expression of the pre-miR92b was decreased only to 55% of the control levels. Similarly, expression of the mature miR107 was decreased to 22% of control levels following UVB exposure, whereas UVB decreased pre-miR107 expression only to 52% of the control levels. In these two cases, although UVB exposure decreased the expression of the pre-miRNA, the subsequent decrease in the mature miRNA expression level was of a higher magnitude. UVB exposure also decreased the expression of miR188 to 28% of the control levels, whereas the expression of pre-miR188 significantly increased to 192% of that in the non-irradiated controls (Fig. 2I).

### The TCF/LEF–β-catenin complex binds to the Dicer promoter and regulates Dicer transcription

**Fig. 3.**
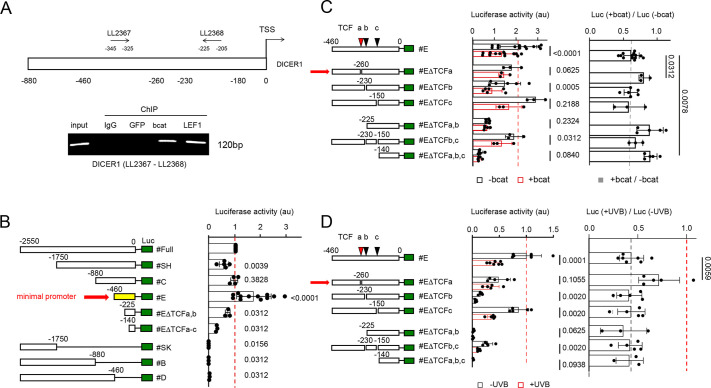
**The TCF/LEF–β-catenin complex directly regulates transcription of Dicer.** (A) ChIP assays were conducted to assess the binding of β-catenin to the Dicer promoter in SW620 cells. The experiments were repeated three times (*n*=3). The schematic (top) shows the Dicer promoter with base pair numbering relative to the TSS. The positions of the oligonucleotides used are marked. The representative gel image (bottom) shows association of β-catenin (bcat) and LEF1 with the DICER promoter in SW620 cells. Rabbit immunoglobulins (IgG) and green fluorescent protein (GFP) were used as negative control antibodies. Input indicates non-immunoprecipitated chromatin. (B) Left: schematic representation illustrating various Dicer promoter luciferase (Luc) constructs with either 5′ or 3′ regions of the Dicer promoter removed. Right: luciferase activities (measured in arbitrary units and expressed relative to activity of the full promoter construct) were assessed in melan-a cells to identify the minimal promoter region (indicated by the horizontal red arrow in the schematic). (C,D) Left: schematics depicting various Dicer minimal promoter constructs with or without three potential TCF/LEF–β-catenin binding sites (represented as colored triangles). (C) Luciferase activities of each construct were measured in the presence or absence of exogenous β-catenin (bcat), revealing that constructs #EΔTCFa, #EΔTCFa,b and #EΔTCFa,b,c exhibit reduced sensitivity to the presence of β-catenin. (D) Luciferase activities of each construct were measured in the presence or absence of UVB irradiation, demonstrating that construct #EΔTCFa loses its sensitivity to UVB exposure. Luciferase activities in C and D are shown relative to the activity of the minimal promoter construct (middle) and as the ratio of activity in treated and untreated cells (right). Data in B–D are presented as mean±s.d. The presented data correspond to experiments that were conducted biologically at least three times with three independent technical replicates for each. Statistical analysis was performed using a Wilcoxon matched-pairs signed-rank test. The *P*-values are given in the figure. Au, arbitrary units. The horizontal red arrow in B shows the minimal promoter necessary for Dicer expression. The horizontal red arrows in C and D indicate the construct that was used to identify the specific TCF/LEF binding site that is necessary for β-catenin (C) and UVB (D)-mediated repression of Dicer expression.

Because β-catenin is a recognized gene expression regulator, we investigated the Dicer promoter for potential TCF/LEF transcription factor binding sites within the initial 300 base pairs upstream of the TSS (transcription start site) using the TRANSFAC database (https://genexplain.com/transfac/). Chromatin immunoprecipitation (ChIP) experiments showed that both β-catenin and LEF1 were associated with the Dicer promoter in SW620 cells (Fig. 3A).

To pinpoint the sequences and/or domains in the Dicer promoter necessary for regulating Dicer transcription, we generated truncated versions of the Dicer::luciferase promoter reporter (Levy et al., 2010). We compared the luciferase activity of the full Dicer promoter with that of different truncated variants in melan-a cells (Fig. 3B). C-terminal deletions of the promoter (constructs #SK, #B and #D) completely abolished luciferase activity, indicating the presence of essential elements near the TSS. Progressive N-terminal deletions of the promoter revealed that 460 base pairs upstream of the TSS still contained crucial elements for promoter activity. Thus, we designated construct #E as the minimal promoter (Fig. 3B). Both the overexpression of β-catenin and UVB irradiation resulted in decreased luciferase activity of the minimal promoter (Fig. 3C,D). Dicer expression is also regulated post-transcriptionally, as a number of alternative splicing variants have been identified for the Dicer transcript, with some encoding shorter Dicer isoforms and others not encoding any protein (Grelier et al., 2009; Hinkal et al., 2011; Kurzynska-Kokorniak et al., 2015). A link might exist between β-catenin and alternative splicing. Indeed, it has been shown in colorectal cancer cell lines that β-catenin regulates expression of the splicing factor SRp20 (also known as SRSF3) (Gonçalves et al., 2008). For now, we have not evaluated the potential role of SRp20 or β-catenin in the alternative splice regulation of Dicer in melanocytes and melanoma, nor whether UVB exposure has an effect on the alternative splicing of Dicer transcripts.

*In silico* analysis of the minimal Dicer promoter sequence using TRANSFAC identified three putative binding sites for TCF/LEF proteins, characterized as MAMAG (where M represents A or C) or CTKTK (where K represents G or T). To investigate the importance of these sites in regulating the Dicer minimal promoter, we generated deletions of the five nucleotides corresponding to these consensus sites (see Materials and Methods). Deletion of the TCFa binding site nearly abolished the β-catenin- and UVB-mediated repression of luciferase reporter activity (Fig. 3C,D; Fig. S2; construct #EΔTCFa), and also decreased the overall activity of the promoter. These observations are consistent with a situation wherein removal of the TCFa site results in loss of recruitment of a particular unknown non-TCF/LEF transcription factor (transcription factor X, TFX) that promotes expression from the promoter. In this scenario, TCF/LEF alone would have a lower binding affinity for the TCFa site than TFX, whereas in the presence of β-catenin, the TCF/LEF–β-catenin complex would have higher affinity for the TCFa site but lower transcriptional activity than TFX, resulting in reduction of Dicer expression.

Individual deletions of the other two sites, TCFb and TCFc, or concomitant deletions of both, did not affect the luciferase activity response of the construct to β-catenin overexpression or UVB irradiation in a significant way. This analysis demonstrated that the repression of the Dicer promoter by β-catenin or UVB is dependent on the presence of TCF/LEF binding sites. Interestingly, previous studies have also suggested that SOX4 promotes Dicer transcription by binding to the Dicer gene promoter (Jafarnejad et al., 2013). Furthermore, SOX4 competes with β-catenin for binding to TCF/LEF proteins in colon carcinoma cells (Sinner et al., 2007), thus acting as a negative regulator of β-catenin transcriptional activity. In this respect, β-catenin and SOX4 might be involved in differentially regulating Dicer expression, at least in the melanocyte lineage.

Dicer is a crucial protein that is involved in several fundamental processes, including embryonic development and cellular stress responses, through the regulation of gene expression and modulation of DNA repair, respectively. As such, understanding the mechanisms regulating its expression is of vital importance. By identifying and characterizing β-catenin as a transcriptional regulator of Dicer following UVB exposure, our work brings to light the importance and intricacy of the network consisting of β-catenin, Dicer, MITF, UVB, cell migration, pigmentation and DNA repair. Moreover, our results underscore the fundamental roles played by UVB, β-catenin and Dicer in establishing, renewing and transforming the melanocyte lineage.

## MATERIALS AND METHODS

### Cell culture and transfection procedures

The melan-a mouse melanocyte cell line was generously provided by Dr Dorothy Bennett (Bennett et al., 1987), and the 9v mouse melanocyte cell line was generated previously (Delmas et al., 2007). These cells were cultured in F12 medium (Gibco) supplemented with 10% fetal calf serum (Gibco) and 200 nM tetradecanoyl phorbol acetate (TPA; Sigma). MNT-1, SK28 and 501Mel human melanoma cells were grown in RPMI-1640 medium supplemented with 10% fetal calf serum (Moore et al., 2004). Murine NIH3T3 fibroblasts and XB2 keratinocytes were maintained in DMEM medium (GIBCO) supplemented with 10% fetal calf serum. SW620 human colon carcinoma cells were grown in Leibovitz's L-15 medium supplemented with 10% fetal calf serum and 2 mM L-glutamine. Cell lines were authenticated using the STR system and were mycoplasma negative. Antibiotics (100 U/ml penicillin and 100 μg/ml streptomycin) were added to all culture media, and cells were incubated at 37°C in a humidified atmosphere with 5% carbon dioxide. The MNT-1, SK28 and 501Mel human melanoma cell lines were provided by Dr Evelyne Coudrier (Institut Curie, Paris, France), Dr Florence Faure (Institut Curie, Paris, France) and Dr Colin Goding (Ludwig Cancer Research Center, Oxford, UK). Murine NIH3T3 fibroblasts and XB2 keratinocytes were provided by Dr Georges Calothy (Institut Curie, Paris, France) and Dr Dorothy Bennett (St. George's, University of London, UK). SW620 colon carcinoma cells were provided by Dr Sylvie Robine (Institut Curie, Paris).

For transient transfection, cells at 70% confluence were transfected using Lipofectamine 2000 (Invitrogen) following the manufacturer's instructions. In luciferase assays, cells seeded in 12-well plates were co-transfected with either 1 μg of total plasmid DNA, along with the TK::Renilla luciferase construct used as a control. The mass of DNA was adjusted to be equal with pBluescript. Firefly and Renilla luciferase activities were assessed 48 h after transfection, unless otherwise specified, using the Dual Luciferase Reporter Assay kit from Promega. Firefly luciferase activity was normalized against Renilla luciferase activity.

For chemical inhibitors, the MEK inhibitor U0126 (9903, Cell Signaling Technology), the PKC inhibitor Enzastaurin (SML0762, Sigma) and the ATM inhibitor KU-55933 (SML1109, Sigma) were all used at 10 μM. The PI3K inhibitor LY294002 (440202, Sigma) was used at 50 μM. The RSK inhibitor BI-D1870 (559286, Sigma) was used at 20μM, and the GSK3β inhibitor BIO (B1686, Sigma) was used at 5 μM. Treatments were done for 6 h, except for KU-55933, which was used for 24 h. The β-catenin-TCF/LEF inhibitor, iCRT3 (SML0211, Sigma), was used at 10 μM for 6 h. The TOP-flash reporter has been previously described (Korinek et al., 1997).

### UV irradiation and sample preparation

UV irradiation was conducted using a VL-330 mid-range lamp with a continuous spectrum ranging from 250 nm to 400 nm, with a peak emission at 313 nm. The dose rate of our UV system underwent calibration by the French National Metrology Institute (LNE France, Laboratoire National de Metrologie et d'Essais). Based on the spectral energy distribution of the UV source, measured at a distance of 23 cm from the lamps, it was determined that 70% of the total radiation fell within the UVB range, 29.9% within the UVA range and a minimal component (0.1%) resided at the upper end of the UVC range. To achieve a dose of 100 mJ/cm^2^ UVB, an exposure duration of 25 s was necessary. To ensure consistent UVB radiation levels, we regularly monitored UVB intensity using an IL1700 radiometer before each exposure, occurring every other week.

For UV irradiation of cell cultures, we first removed the medium from cultures when they reached 80% confluence. Subsequently, we washed the cells twice with phosphate-buffered saline (PBS) and replaced the medium with fresh PBS. During UV irradiation, the lid of the plastic dish was removed. Following UV exposure, we substituted the PBS with fresh culture medium, and the cells were then incubated for varying durations, depending on the specific experimental requirements. Control cultures underwent a similar treatment process but omitted the UV exposure step.

### RNA extraction and RT-qPCR analysis

To extract total RNA from cultured cell lines, we utilized the RNeasy mini kit (Qiagen), following the manufacturer's guidelines. The recovered RNA was quantified using a Nanovue device (General Electric).

Reverse transcription reactions were conducted using 1 μg of total RNA and M-MLV reverse transcriptase (Invitrogen), following the manufacturer's protocols. The resulting cDNA was then subjected to semi-quantitative real-time PCR analysis of gene expression. This reaction was performed in a QuantStudio real-time PCR System. We estimated the quantity of Dicer and different housekeeping gene mRNAs using the appropriate oligonucleotides (Table S1) and SYBR Green Supermix (Bio-Rad). The thermal profile consisted of an initial denaturation step at 95°C for 90s, followed by 40 cycles of denaturation at 95°C for 30s and annealing/extension at 60°C for 1 min. The obtained results for *Dicer* mRNA expression were normalized to the expression of *Gapdh* or *Hprt* (for mouse cells), or to the expression of *TBP*, *GAPDH* or *SDHA* (for human cells), which served as housekeeping genes. The relative amount of the target transcript in comparison to the reference gene transcript was determined using the ΔΔCt method (Livak and Schmittgen, 2001). Each experiment was repeated a minimum of three times, and the results from triplicate experiments are presented for each time point. Representative amplification plots and melting curves for each primer sets are presented in Fig. S3A.

For analysis of miR levels, reverse transcription reactions were conducted using the miRCURY LNA RT kit (339340, Invitrogen), following the manufacturer's protocols. The resulting cDNA was used in miRCURY LNA miRNA PCR assays specific to each miRNA of interest (miR92b, miR107 and miR188) and a normalization reference (RNU5B-1). The reactions were performed in a QuantStudio real-time PCR System. All raw data are given in Table S2.

### Western blotting

To initiate western blotting, cells were lysed in ice-cold RIPA buffer (10 mM Tris-HCl pH 8, 150 mM NaCl, 1% NP-40, 0.5% sodium deoxycholate and 0.1% SDS) enriched with a complete protease inhibitor cocktail and PhoStop phosphatase inhibitor cocktail (both Roche, France). The lysates were then cleared of cell debris by centrifugation at 20,000 ***g*** for 15 min. The resulting supernatants were collected, and their protein concentrations were determined using a BCA assay (Thermo Fisher Scientific). Subsequently, 50–100 μg of total protein was separated on a denaturing 10% acrylamide SDS-PAGE gel through electrophoresis.

Following electrophoretic separation, proteins were transferred onto a nitrocellulose membrane (Amersham), which was further treated with Ponceau Red solution to assess the quality of the transfer. To block non-specific binding, the membrane was incubated for 1 h in a solution containing 5% non-fat milk powder (Regilait) in Tris-buffered saline supplemented with 0.01% Tween-20 (TBST). The membrane was then subjected to an overnight incubation with primary antibodies, dissolved in a 5% non-fat milk powder TBST solution. Afterward, the membrane underwent three rounds of washing with TBST.

Subsequently, the membrane was incubated with the corresponding horseradish peroxidase (HRP)-conjugated secondary antibodies at a 1:10,000 dilution. Following another set of three TBST washes, antibody binding was detected using enhanced chemiluminescence (ECL; Thermo Fisher Scientific, France). The list of primary antibodies used is provided in Table S1 and includes details of antibodies used to detect Dicer, vinculin, actin, phosphorylated ERK (p204-ERK), ERK, phosphorylated GSK3β (p9-GSK3β), GSK3β, phosphorylated CHK2 (p68-CHK2), CHK2, phosphorylated AKT (p473-AKT), AKT, phosphorylated β-catenin (p33,37,41-β-catenin), and β-catenin. Primary antibody dilutions were shown in Table S1. All experiments were independently conducted at least three times. Western blot data were quantified using ImageJ (NIH, Bethesda, USA) and are presented in Table S2. Raw blot data are given in Fig. S3B and Fig. S4.

### Chromatin immunoprecipitation

ChIP experiments were performed as previously described (Aktary et al., 2013). The composition of all buffers is provided in Table S1. Confluent cultures in 150 mm dishes were trypsinized, and 2×10^7^ cells pelleted by centrifugation at 1300 ***g*** for 10 min. The cell pellets were then resuspended in growth medium to which formaldehyde (Fisher) was added to a final concentration of 1% and incubated at room temperature for 10 min. To stop fixation, glycine was added to a final concentration of 125 mM. The cell suspension was then centrifuged at 1300 ***g*** at 4°C for 10 min. The resulting cell pellets were then washed twice with PBS containing 1 μg/ml aprotinin and leupeptin (Sigma) and 1 mM PMSF (Sigma), after which they were resuspended in cell lysis buffer and incubated on ice for 15 min. NP-40 (Sigma) was then added (final concentration of 0.6%), after which the samples were vortexed for 10 s at high speed and subsequently centrifuged at 18,000 ***g*** for 30 s. The resulting pellets were then resuspended in sonication buffer (Table S1) and left on ice for 10 min. The samples were then sonicated (Branson Sonifier 450) for 1 min at 20% output for a total of four times.

The sonicated chromatin samples were then diluted ten-fold in chromatin dilution buffer (Table S1) after which 50 μl was removed (Input). Then, 40 μl Protein A/G Agarose beads (Calbiochem) were added and the samples were pre-cleaned on a rocker-rotator at 4°C for 2 h. Following incubation, the samples were centrifuged briefly (2600 ***g*** for 5 min at 4°C), and the resulting supernatant (pre-cleaned chromatin) was split into equal aliquots and processed for immunoprecipitation. Each aliquot was incubated with 5 μg antibodies and 40 μl pre-cleaned [by overnight incubation with 4 μg salmon sperm DNA (Thermo Fisher Scientific) and 5 μg BSA (Sigma)] Protein A/G Agarose beads overnight at 4°C on a rocker-rotator. Antibody details are given in Table S1.

Following immunoprecipitation, the samples were centrifuged for 10 min at 425 ***g*** at 4°C, after which the resulting supernatants were removed. The beads were then subjected to six 5 min washes in each of four wash buffers (Table S1). Following the washes, the protein–DNA complexes were eluted off the beads by incubation in elution buffer for 15 min at room temperature on a rocker-rotator.

Following elution, 1 μg RNase (Sigma) and NaCl (final concentration 300 mM) were added to the samples, which were then incubated at 65°C for 4 h. Next, Tris-HCl pH 6.8, EDTA (final concentrations of 40 mM and 10 mM, respectively) and 4 μg proteinase K (Roche) were added to the samples, which were incubated at 45°C for 2 h. The samples were then purified using a PCR Purification Kit (QIAGEN, Valencia, CA, USA) and processed for PCR.

### Plasmid constructs

The wild-type Dicer::luciferase promoter was kindly provided by Dr David Fisher (Massachussetts General Hospital, Boston, USA) and has been described elsewhere (Levy et al., 2010). The constructs #B, #C, #D, #E, #SH, #SK and #EΔTCFa,b,c of the Dicer promoter were generated by digesting the full Dicer::luciferase promoter with the following restriction enzymes: KpnI and NcoI (#B), NcoI and HindIII (#C), KpnI and EcoRI (#D), EcoRI and HindIII (#E), SmaI and HindIII (#SH), SmaI and KpnI (#SK), SacII and HindIII (#EΔTCFa,b,c). The resulting fragments were eluted on agarose gels and inserted into the pGL3 luciferase vector (Promega) digested by SmaI and KpnI or by SmaI and HindIII.

The constructs #EΔTCFa, #EΔTCFb, #EΔTCFc and #EΔTCFa,b of the Dicer promoter were generated by PCR-mediated deletion mutagenesis by overlap extension PCR in two steps to remove the putative binding site sequences: TCFa, CTTTC; TCFb, CACAG; TFCc, CTGTG. The oligonucleotides list is shown in Table S1. All raw data are given in Table S2.

The β-catenin overexpression construct (CMV-bcat*) has been previously described (Delmas et al., 2007).

## Supplementary Material



10.1242/joces.261978_sup1 Supplementary information

Table S1.

Table S2.
