# Supplementary material for: UVB radiation suppresses Dicer expression through β-catenin
Source: J Cell Sci. 2024 Nov 26;137(22):jcs261978. doi: 10.1242/jcs.261978 (PMC11634033; doi:10.1242/jcs.261978)
Supplement: Supplementary information [file joces-137-261978-s1.pdf]

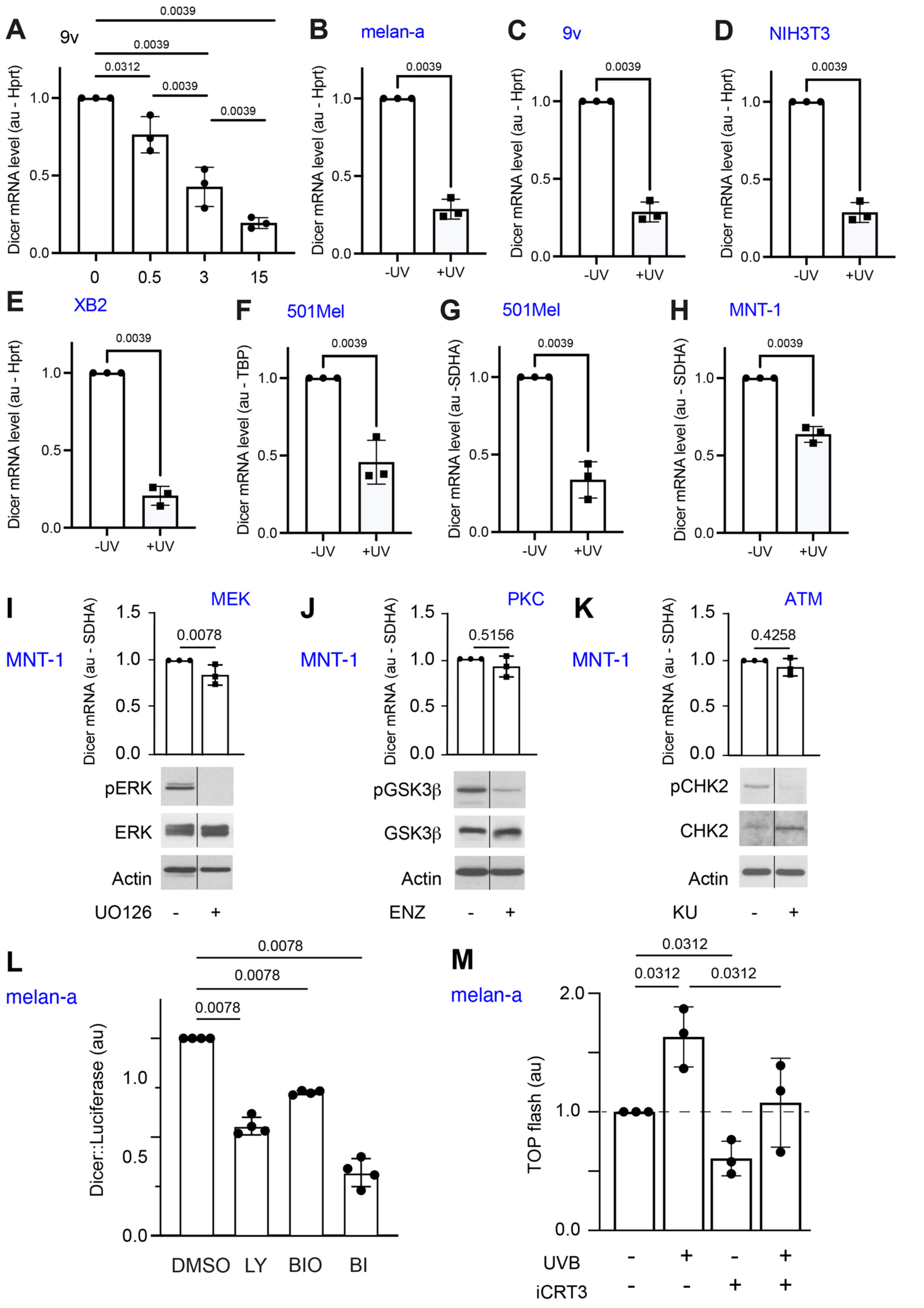

Journal of Cell Science • Supplementary information

Journal of Cell Science • Supplementary information

Fig. S1. Modification of Dicer level after UVB-irradiation and modification of signaling pathways

(A-H) UVB-irradiation represses Dicer expression in various cell types. Dicer transcript levels were assessed using RT-qPCR following UV irradiation or without UV exposure. (A) RNAs were extracted from 9v cells 0-15 hours after UVB irradiation at 100 mJ/cm^2. (B-H) RNAs were collected 15 hours after UVB irradiation at 100 mJ/cm^2 (+UV) or without irradiation (-UV) mouse cell lines, melan-a (B) and 9v (C) melanocytes, NIH3T3 fibroblasts (D), and XB2 keratinocytes (E). Similar experiments were performed with 501Mel (F,G) and MNT-1 (H) human melanoma cells after UVB irradiation at 25 mJ/cm^2. The readouts were normalized to Hprt for mouse (A-E), to TBP (F) and SDHA (G,H) for human cell lines. Note that in our conditions, TBP was sensitive to UVB whereas GAPDH and SDHA were not. However, the sensitivity of Dicer to UV was more drastic than TBP.

(I-M) Effect of various signaling pathways on Dicer expression. The effect of various signaling pathways on the expression of Dicer in MNT-1 cells was assessed using different chemical inhibitors. (I-K) The MEK inhibitor, U0126 (I), the PKC inhibitor, Enzastaurin (J), and the ATM inhibitor, KU-55933 (K), were all used at 10µM. Treatments were done for 6 hours, except for KU-55933, which was used for 24 hours. The effectiveness of each inhibitor was validated by assessing the phosphorylation levels of downstream signaling proteins. (L) Effect of LY294002, BIO and BI-D1870 on Dicer expression using a Dicer::luciferase reporter construct (Levy et al., 2010) following a 24 hour treatment with each inhibitor in melan-a cells. (M) The efficacy of iCRT3, the β-catenin-Tcf inhibitor, was validated in melan-a cells by assessing TOP-flash activity (Korinek et al., 1997) 6 hours after treatment with 10µM iCRT3.

The presented data correspond to experiments that were conducted biologically at least three times with three independent technical replicates for each. Statistical analysis was performed using a Wilcoxon matched-pairs signed rank test. Bars represent means ± sd. The p-value are given on the panels.

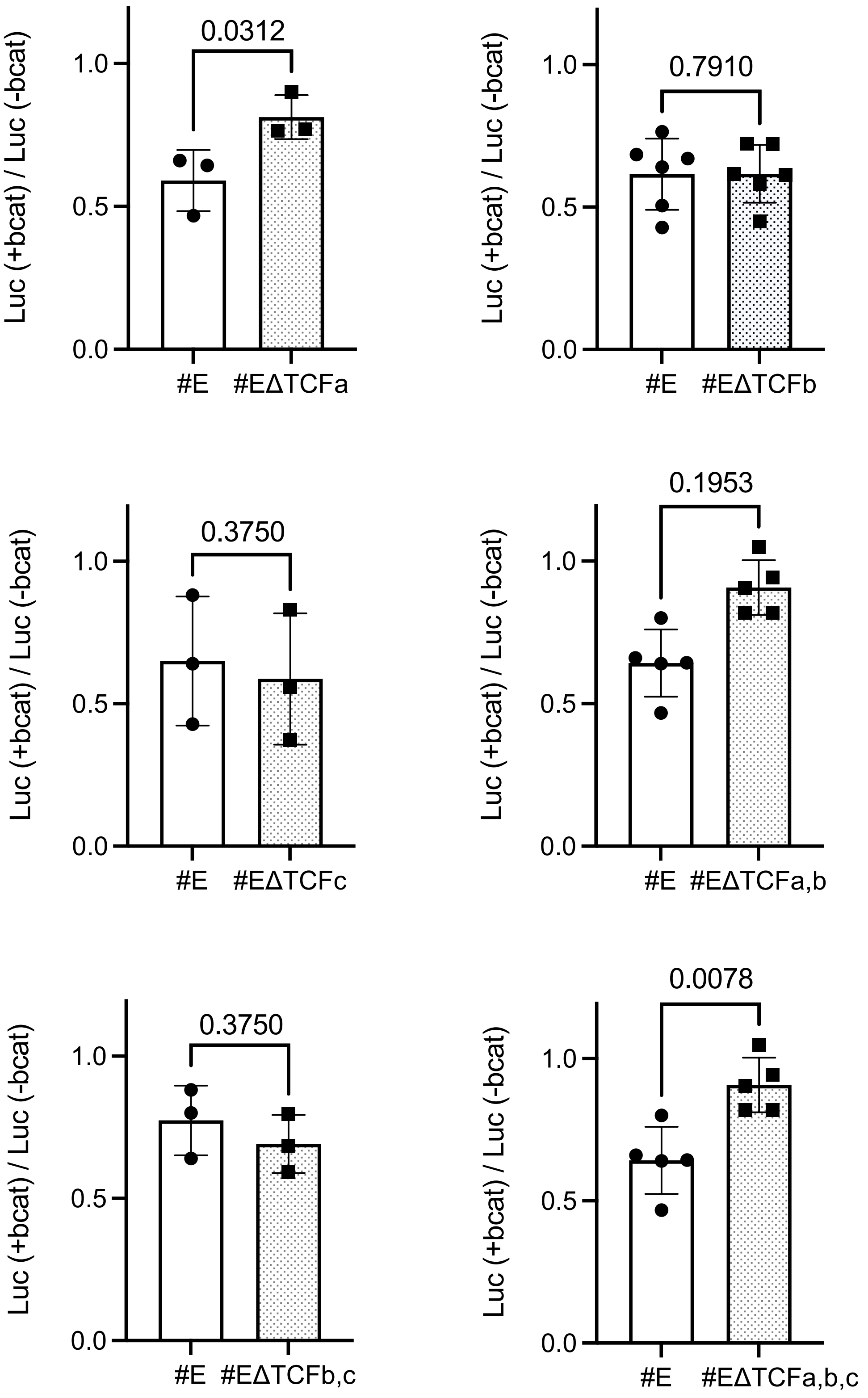

Fig. S2. Site TCFa is essential for β-catenin-mediated repression of Dicer

Luciferase activities were measured in the presence or absence of exogenous β-catenin (bcat), revealing that constructs #EΔTCFa, #EΔTCFa,b, and #EΔTCFa,b,c exhibit reduced sensitivity to the presence of b-catenin. The presented data correspond to experiments that were conducted biologically at least three times with three independent technical replicates for each. Statistical analysis was performed using a Wilcoxon matched-pairs signed rank test. Bars represent means ± sd. The p-value are given on the panels.

Journal of Cell Science • Supplementary information

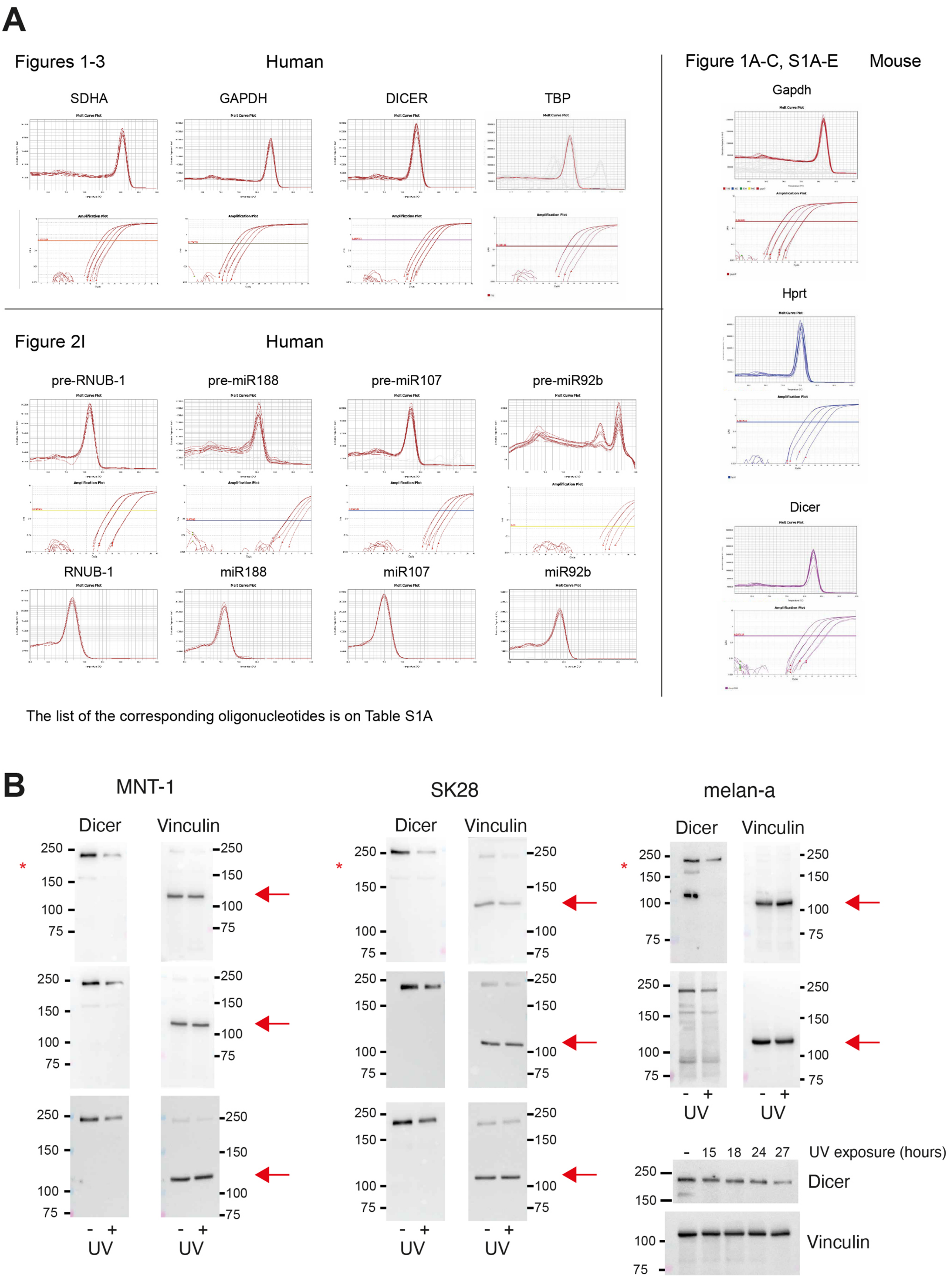

Fig. S3. Amplification and melting curves for various primers used in this study (A) and triplicates of Western blots revealing Dicer and Vinculin in various cell lines (B)

Journal of Cell Science • Supplementary information

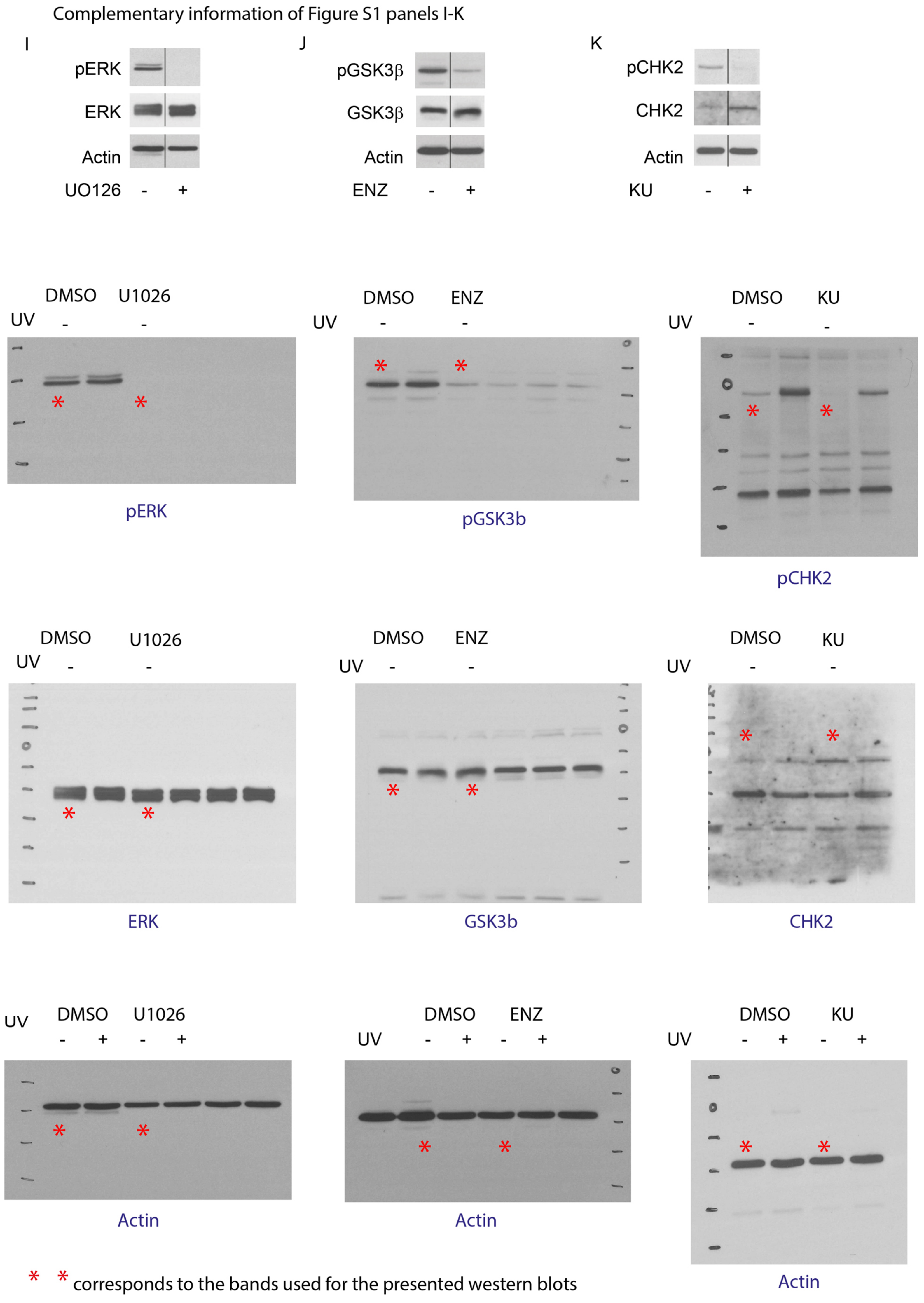

Fig. S4. Blot transparency for data shown in Fig. S1.

Journal of Cell Science • Supplementary information

Table S1.

Available for download at
https://journals.biologists.com/jcs/article-lookup/doi/10.1242/jcs.261978#supplementary-data

Table S2.

Available for download at
https://journals.biologists.com/jcs/article-lookup/doi/10.1242/jcs.261978#supplementary-data
