# Supplementary material for: UVB radiation suppresses Dicer expression through β-catenin
Source: J Cell Sci. 2024 Nov 26;137(22):jcs261978. doi: 10.1242/jcs.261978 (PMC11634033; doi:10.1242/jcs.261978)
Supplement: Supplementary file 1 [file joces-137-261978_review_history.pdf]

Journal of Cell Science

UVB radiation suppresses Dicer expression through β-catenin

Zackie Aktary, Valerie Petit, Irina Berlin, Jeremy Raymond, Frederique Berger, Nisamanee Charenchaon, Evelyne Sage, Juliette Bertrand and Lionel Larue
DOI: 10.1242/jcs.261978

Editor: Kathleen Green

Review timeline

Original submission:	24 January 2024
Editorial decision:	11 March 2024
First revision received:	17 July 2024
Editorial decision:	9 September 2024
Second revision received:	1 October 2024
Accepted:	7 October 2024

Original submission

First decision letter

MS ID#: JOCES/2024/261978

MS TITLE: UVB radiation suppresses Dicer expression through β-catenin

AUTHORS: Zackie Aktary, Valerie Petit, Irina Berlin, Nisa Charenchaon, Evelyne Sage, Juliette U. Bertrand, and Lionel Larue

We have now reached a decision on the above manuscript.

To see the reviewers' reports and a copy of this decision letter, please go to: https://submit-jcs.biologists.org and click on the 'Manuscripts with Decisions' queue in the Author Area. (Corresponding author only has access to reviews.)

As you will see, the reviewers raise a number of substantial criticisms that prevent me from accepting the paper at this stage. They suggest, however, that a revised version might prove acceptable, if you can address their concerns. If you think that you can deal satisfactorily with the criticisms on revision, I would be pleased to see a revised manuscript. We would then return it to the reviewers.

Please ensure that you clearly highlight all changes made in the revised manuscript. Please avoid using 'Tracked changes' in Word files as these are lost in PDF conversion.

I should be grateful if you would also provide a point-by-point response detailing how you have dealt with the points raised by the reviewers in the 'Response to Reviewers' box. Please attend to all of the reviewers' comments. If you do not agree with any of their criticisms or suggestions please explain clearly why this is so.

Reviewer 1

Advance summary and potential significance to field

© 2024. Published by The Company of Biologists under the terms of the Creative Commons Attribution License (https://creativecommons.org/licenses/by/4.0/).

In their manuscript, Akatary et al studied the regulation of DICER by UVB radiation. They demonstrate that UVB inhibits DICER expression in various cell types. This effect is transcriptional and mediated by the LEF/β-Catenin complex.
Given the central role of DICER in cell biology, and its regulation by numerous stresses, it is of paramount importance to describe the regulation of DICER by UVB. Indeed, UVB is one of the most harmful compulsory environmental stimuli responsible for a wide range of skin diseases, including cancer and premature skin aging conditions.
The paper is globally well written, and the technical approaches appropriated.
However, I have noticed a few errors and improvements are possible.

Comments for the author

1) in the introduction, a lot of information is focused on melanocytes.
however, keratinocytes and fibroblasts are also exposed to UV radiation from sunlight. It is therefore important in the introduction to present what we know about the effects of UV on these two cell types.
2) Figure 1 is mislabeled, panel F is E. Could the authors quantify the western blots to assess % inhibition of DICER expression?
3) The legend on figure 2 is a bit confusing. Could you please clarify the treatments.
4) Does BIO inhibitor also reverse the effect of UVB on dicer?
5) Can the authors comment on the possible effect of WNT ligands on DICER and on the effect of UV on MITF expression
6) Can the authors comment on the specificity of action of BIO and iCRT3 inhibitors?
7) TCF binding site A appears to be the most important site in mediating the inhibitory effect of UV on the DICER promoter. However, elimination of this site also decreases the overall activity of the promoter. Can the authors comment on this point?

Reviewer 2

Advance summary and potential significance to field

The authors conclude that treatment of several different mouse and human cell types in vitro with UV reduces the expression of Dicer by about two fold due to transcriptional repression by B-catenin. Since the down-regulation of Dicer by UV has been previously demonstrated in mouse preadipocytes, the advances here are demonstration of this relationship in human cells and more cell types, including those of the skin which are frequently exposed to UV; as well as the mechanistic link to B-catenin.

Comments for the author

The manuscript presents a series of in vitro experiments designed to query the mechanisms by which UV exposure reduces Dicer expression. If the observations are clarified and substantiated, the manuscript should be considered for publication. However, the manuscript in its present form suffers from a lack of transparency, assay validation, and clarity. Below are a series of requirements for reconsideration of the manuscript. Most are requiring only more transparency in the form of plotting raw and individual data points, images, quantification of existing data, statistics, etc, and therefore should not be overly burdensome. A second broad concern is the lack of experiments that demonstrate that pathway activation increases Dicer expression and assays for the expected downstream effects of changing Dicer levels. These (points 5 and 6) will require additional experiments, but given the simple in vitro systems and assays being employed, are not expected to be overly burdensome in time or cost.

1. More transparency of experiments in figures and legends
a. RT-qPCR: include in axis label and legend what housekeeping gene is used.
b. Methods state GAPDH is the reference gene. Is this true for both mouse and human cells? Table S1 suggests the GAPDH genes are mouse specific.
c. Figs 1A, B and all western blots lack statistics. The Figure 1 legend states all experiments were conducted at least three times, but Figs 1B and D show no indication of variance. Lack of appropriate statistics is unacceptable and must be fixed.

© 2024. Published by The Company of Biologists under the terms of the Creative Commons Attribution License (https://creativecommons.org/licenses/by/4.0/).

d. For all experiments, plot the individual data points in addition to the mean, unless well justified.
e. For all experiments plot standard deviation instead of SEM, unless well justified.
f. For the sake of transparency – can the authors confirm, both here in their response as well as in the manuscript methods and figure legends, that the unpaired student t-tests were run using the raw, non-normalized, values? Panels such as Fig 1 A-C and all of Figure 2 all include a normalized control bar where the value was set to '1' for all experiments with an SEM of '0'. That is fine for visualization, but it is inappropriate to use these values for the statistics as the practice artificially erases variance of the control.
g. Provide the source data (raw and normalized) for all graphs in supplemental table format.

2. The authors need to verify the fidelity of the luciferase reporter by demonstrating that luciferase expression tracks with Dicer expression in a system where differential Dicer expression is already established. Similarly the authors need to include a luciferase construct for a different (non-Dicer) promoter and demonstrate it does not respond to UV/bcat, etc. These are basic controls.

3. GAPDH is a curious choice of a housekeeping gene. It is acknowledged that it is frequently used for this purpose, but regardless, it is prudent to consider the system you are using. In this case, the expression of GAPDH is regulated by both Pi3K signaling and p53 (reviewed in PMID: 25859407). Might the reduction in relative Dicer be the consequence of an increase in GAPDH? For all RT-qPCR data:
a. Try a second housekeeping gene that is not regulated by the pathways being queried.
b. Try a second experimental gene besides Dicer, this is not regulated by the pathways being queried to determine whether its expression appears stable when made relative to GAPDH
c. Provide the raw data – including representative amplification and melting curves as well as the Ct values for each experiment, so that it can be assessed by the reader. These are just the minimal requirements of the MIQE guidelines (PMID: 19246619)
d. A consistent housekeeping loading control would strengthen the manuscript – as is, it is not clear what loading control is used for which experiment (GAPDH, TBP, Actin, etc), but whatever loading controls the authors use, it is necessary to show that their expression, both RNA and protein, are not affected by UV dosing – similar to Fig. 1A and B.
e. To be clear – much of this manuscript hinges on the interpretation of RT-qPCR data. In almost all panels, the RT-qPCR data presented show a 2x change in signal, which is the equivalent of a single cycle / Ct. In some cases (Fig 2H) the authors claim $p<0.001$ significance between a relative RT-qPCR value of 1 compared to 1.3 – which is less than one Ct difference and thereby less than the theoretical sensitivity of the assay. The authors, therefore, are required to provide transparency and quality control of their RT-qPCR to present a convincing argument. I will reiterate that transparency of both the assay/data as well as the statistics (as discussed in point 1) are required.

4. Figure 1D, as is, does not support the interpretation that 'the level of Dicer protein was decreased across transformed and non-transformed cell lines.'
In particular, the melan-a decrease is non-obvious and the associated actin bands do not appear to match. The methods state that western blots were all conducted at least three times. It is necessary to provide these data in the form of both raw scans and semi-quantification.

5. Related to Figs 2 and 3: The authors need to demonstrate that activation of P13K, RSK and GSK3B also increase Dicer expression and promoter activity, as would be expected if their hypothesis were true.

6. The authors should measure the pre-miRNA and mature miRNA for well-expressed miRNAs in the +/- UV and pathway inhibitor and activating conditions.
If their hypothesis were accurate, an increase in pre and a decrease in mature would be expected and would verify the presented results.

Minor:
7. The text indicates that Dicer mRNA was reduced after UV exposure in WM852 cells, citing Fig. 1C, but there are no data to support this claim.

© 2024. Published by The Company of Biologists under the terms of the Creative Commons Attribution License (https://creativecommons.org/licenses/by/4.0/).

8. Figure 1 “F”, should be Fig 1 “E”

First revision

Author response to reviewers' comments

Reviewer 1 Advance Summary and Potential Significance to Field:

In their manuscript, Akatary et al studied the regulation of DICER by UVB radiation. They demonstrate that UVB inhibits DICER expression in various cell types. This effect is transcriptional and mediated by the LEF/β-Catenin complex. Given the central role of DICER in cell biology, and its regulation by numerous stresses, it is of paramount importance to describe the regulation of DICER by UVB. Indeed, UVB is one of the most harmful compulsory environmental stimuli, responsible for a wide range of skin diseases, including cancer and premature skin aging conditions. The paper is globally well written, and the technical approaches appropriated. However, I have noticed a few errors and improvements are possible.

Reviewer 1 Comments for the Author:

R1 Q1)in the introduction, a lot of information is focused on melanocytes. However, keratinocytes and fibroblasts are also exposed to UV radiation from sunlight. It is therefore important in the introduction to present what we know about the effects of UV on these two cell types.

R1 A1) The referee is right. In the introduction we added the following: “*Cells of the skin, including melanocytes, keratinocytes, and dermal fibroblasts, are continuously exposed to the potentially harmful effects of ultraviolet radiation (UV) from sunlight, which can lead to DNA damage, oxidative stress, inflammation, and changes in gene expression. To mitigate these risks, this organ has developed a variety of defense mechanisms. Different cell types employ both general and specific strategies for defense. General protective measures include processes such as cell cycle inhibition, replication suppression, DNA repair, and apoptosis. Specifically, keratinocytes produce a-MSH, which signals to melanocytes to increase melanin production, while fibroblasts generate more metalloproteinases (MMPs), which break down collagen and elastin fibers—essential components of the extracellular matrix.*”

R1 Q2)Figure 1 is mislabeled, panel F is E. Could the authors quantify the western blots to assess % inhibition of DICER expression?

R1 A2) We thank the referee for pointing out this mistake. It is now fixed.

For Figure 1D, the referee is right. We have now quantified our western blot data and added the statistics. As mentioned by the second reviewer, the western blots were not profoundly convincing for an external eye. We therefore acted on this issue; besides changing the batch of antibodies, we improved the protocols to optimize the relative differences of expression of Dicer and observed that 27 hours after irradiation, decreases in the levels of Dicer were more apparent. The reduction of the level of Dicer protein is around 50%. The different western blots are shown and presented as Western blot transparency.

R1 Q3)The legend on figure 2 is a bit confusing. Could you please clarify the treatments.

R1 A3) We have modified the figure legend to clarify. Additionally, certain panels from the old Figure 2 are now found in Supplementary Figure 2. The new legend for Figure 2 reads, “*The effect of various signaling pathways on the expression of Dicer in MNT-1 cells was assessed using different chemical inhibitors. (A-C) The PI3K inhibitor, LY294002 (A), was used at 50μM. The RSK inhibitor, BI-D1870 (B), was used at 20μM, and the GSK3B inhibitor, BIO (C), was used at 5μM. The effectiveness of each inhibitor was validated by assessing the phosphorylation levels of downstream signaling proteins. All chemical inhibitor treatments lasted 6 hours. (D) Effect of LY294002, BIO and BI-D1870 on Dicer expression using a Dicer::luciferase reporter construct (Levy*

© 2024. Published by The Company of Biologists under the terms of the Creative Commons Attribution License (https://creativecommons.org/licenses/by/4.0/).

et al., 2010) following a 24-hour treatment with each inhibitor. (E) The efficacy of iCRT3, the β-catenin-Tcf inhibitor, was validated by assessing TOP-flash activity (Korinek et al., 1997) 6 hours after treatment with 10μM iCRT3. (F-G) Effect of 6h treatment of UVB (100 mJ/cm^2) in conjunction with either 10μM iCRT3 (F) or 5μM BIO (G) on Dicer mRNA levels as assessed by RT-qPCR. (H) Effect of 6h UVB (100 mJ/cm^2) treatment and/or β-catenin expression on Dicer luciferase transcriptional activity. (I) Effect of 27h UVB (100 mJ/cm^2) on the levels of selected pre-miR and miRNAs."

Moreover, we modified the figure, we included the old panels A-C as supplemental figure. The legend of these three panels are now in supplemental information and Fig. S2A-C.

R1 Q4)Does BIO inhibitor also reverse the effect of UVB on dicer?

R1 A4) We determined the level of Dicer mRNA after treatment of the cells with BIO in the presence and absence of UV irradiation. BIO is an inhibitor of GSK3β, which results in increased β-catenin levels, which in turn should reduce Dicer expression. According to the literature and our data, we did not expect BIO to revert the effect of UV on Dicer levels. In agreement with this hypothesis, we obtained a reduction of the level of Dicer mRNA following treatment with BIO. However, we did not observe a reduction of Dicer in the presence of UV and BIO together compared to UV alone. We did not observe a significant difference between BIO and BIO+UV. This is now shown as Figure 2G.

R1 Q5)Can the authors comment on the possible effect of WNT ligands on DICER, and on the effect of UV on MITF expression

R1 A5) Here, we have demonstrated that β-catenin governs the transcription of Dicer following UV irradiation. Consequently, one might infer that activation of the Wnt pathway could suppress Dicer. Despite our search in the literature, we could not find any publication demonstrating that Wnt ligands regulate Dicer. However, as previously mentioned, β-catenin has been shown to regulate Dicer expression in ovarian cancer cells (To et al., 2017).

UV irradiation has been shown to stimulate Mitf activity through multiple signaling pathways, including the MAPK pathway (Galibert et al., 2001) (Tadokoro et al., 2005). Based on our findings, it is conceivable that UV irradiation induces Mitf via the β-catenin pathway, given that β-catenin has the potential to trigger M-Mitf expression. Moreover, one may think of a negative feedback from Dicer to β-catenin since it has been shown in Xenopus or in mice that Dicer may affect β-catenin gene products production (Wu et al., 2019; Tagami et al., 2021).

We accordingly added a comment on this issue in the "results and discussion" section.

R1 Q6)Can the authors comment on the specificity of action of BIO and iCRT3 inhibitors?

R1 A6) BIO and iCRT3 are both small molecules that target specific components of the Wnt signaling pathway, albeit with different specificities. BIO is a glycogen synthase kinase 3 (GSK3) inhibitor. GSK3β plays a crucial role in the Wnt pathway by phosphorylating β-catenin, marking it for degradation. By inhibiting GSK3β, BIO prevents the degradation of β-catenin, leading to its accumulation in the cytoplasm and subsequent translocation into the nucleus. In the nucleus, β-catenin interacts with different transcription factors and regulates gene expression.

On the other hand, iCRT3 selectively disrupts the interaction between β-catenin and its transcriptional coactivator. This prevents the formation of a functional transcriptional complex necessary for the regulation of Wnt target genes. In terms of specificity, BIO primarily targets GSK3β, while iCRT3 specifically disrupts the interaction between β-catenin and its cotranscriptional regulators (e.g. TCF). Therefore, BIO's specificity lies in inhibiting the activity of GSK3β, whereas iCRT3's specificity lies in disrupting a particular protein-protein interaction within the Wnt pathway. Each compound offers a different approach to modulating Wnt signaling, with potential implications for research and therapeutic development. With respect to β-catenin's signaling/transcriptional activity, BIO promotes β-catenin signaling, whereas iCRT3 inhibits β-catenin signaling mediated by TCF/LEF.

© 2024. Published by The Company of Biologists under the terms of the Creative Commons Attribution License (https://creativecommons.org/licenses/by/4.0/).

We briefly added these points in the text.

R1 Q7)TCF binding site A appears to be the most important site in mediating the inhibitory effect of UV on the DICER promoter. However, elimination of this site also decreases the overall activity of the promoter. Can the authors comment on this point?

R1 A7) Indeed, site A is important to drive the general activity of the Dicer promoter (Figure 3). The specificity of the repression of Dicer by both ß-catenin and UVB is mainly restricted to site A. At this point, it is difficult to answer the referee. One may imagine that the removal of site A would result in the loss of recruitment of a particular transcription factor, X (TFX), besides TCF, that promotes the expression of the promoter. TCF would have a lower binding affinity than TFX. In the presence of ß-catenin, the TCF-ß-catenin complex would have a higher affinity for the site A than TFX, but would have a lower transcriptional activity, resulting in reduction of Dicer expression.

We added this comment in the discussion.

Reviewer 2 Advance Summary and Potential Significance to Field:

The authors conclude that treatment of several different mouse and human cell types in vitro with UV reduces the expression of Dicer by about two fold due to transcriptional repression by B-catenin. Since the down-regulation of Dicer by UV has been previously demonstrated in mouse preadipocytes, the advances here are demonstration of this relationship in human cells and more cell types, including those of the skin which are frequently exposed to UV; as well as the mechanistic link to B-catenin.

Reviewer 2 Comments for the Author:

R2 Q1)The manuscript presents a series of in vitro experiments designed to query the mechanisms by which UV exposure reduces Dicer expression. If the observations are clarified and substantiated, the manuscript should be considered for publication. However, the manuscript in its present form suffers from a lack of transparency, assay validation, and clarity. Below are a series of requirements for reconsideration of the manuscript. Most are requiring only more transparency in the form of plotting raw and individual data points, images, quantification of existing data, statistics, etc, and therefore should not be overly burdensome.

R2 A1) We thank the referee for this comment. On our side, we did not want to lack transparency and clarity. Since we submitted this publication as a *Short report*, we wished to be as brief as possible. We acted on the different concerns raised by the referee, as described below.

R2 Q2) A second broad concern is the lack of experiments that demonstrate that pathway activation increases Dicer expression and assays for the expected downstream effects of changing Dicer levels. These (points 5 and 6) will require additional experiments, but given the simple in vitro systems and assays being employed, are not expected to be overly burdensome in time or cost.

R2 Q2-1 More transparency of experiments in figures and legends
R2 Q2-1 a. RT-qPCR: include in axis label and legend what housekeeping gene is used.

R2 A2-1 a) The housekeeping gene used for each experiment has been added to the legend and axis labels.

R2 Q2-1 b. Methods state GAPDH is the reference gene. Is this true for both mouse and human cells? Table S1 suggests the GAPDH genes are mouse specific.

R2 A2-1 b) We have now incorporated the sequences of the reference genes for both mouse and human into Table S1. In the Materials and Methods section, we specified that Gapdh and Hprt were utilized for mouse samples, while GAPDH, TBP, and SDHA were employed for human samples. We observed that TBP exhibited sensitivity to UV, whereas neither GAPDH nor SDHA did under our experimental conditions. However, the sensitivity of Dicer to UV was more drastic than TBP. We made a note on this in the legend of Fig. S1, but we may remove this point if the editor wishes.

© 2024. Published by The Company of Biologists under the terms of the Creative Commons Attribution License (https://creativecommons.org/licenses/by/4.0/).

R2 Q2-1 c. Figs 1A, B and all western blots lack statistics. The Figure 1 legend states all experiments were conducted at least three times, but Figs 1B and D show no indication of variance. Lack of appropriate statistics is unacceptable and must be fixed.

R2 A2-1 c) We have now quantified the western blots and added the appropriate statistical analyses. For Figure 1B, the lack of error bars in the initial figure was an oversight. This has been corrected.

R2 Q2-1 d.For all experiments, plot the individual data points in addition to the mean, unless well justified.

R2 A2-1 d) All individual data points have been plotted.

R2 Q2-1 e.For all experiments plot standard deviation instead of SEM, unless well justified.

R2 A2-1 e) The standard deviations have now been plotted.

R2 Q2-1 f.For the sake of transparency - can the authors confirm, both here in their response as well as in the manuscript methods and figure legends, that the unpaired student t-tests were run using the raw, non-normalized, values? Panels such as Fig 1 A-C and all of Figure 2 all include a normalized control bar, where the value was set to '1' for all experiments with an SEM of '0'. That is fine for visualization, but it is inappropriate to use these values for the statistics as the practice artificially erases variance of the control.

R2 A2-1 f) We had performed our statistical analyses using the normalized data. However, we do understand the concern of the referee. For that reason, we reanalyzed our data using the unpaired t-test with Welch's correction. This correction accommodates for unequal variance between groups. Overall, this correction changed the p-value of our results, but it did not change the significance of the results. We have to moderate this conclusion for one case: Figure 1B when we tested the dose increment between 10 and 25 mJ/cm^2, since the unpaired t-test p-value was close to 0.05, the Welch's test indicated that the results were not significant.

For information, experiments were conducted using at least three independent biological samples, each with two/three technical replicates. Naturally, the quantities of mRNA and resultant cDNA can fluctuate between biological experiments, potentially leading to artificial differences devoid of biological significance. For this reason, we chose to show normalized values.

R2 Q2-1 g.Provide the source data (raw and normalized) for all graphs in supplemental table format.

R2 A2-1 g) These data are now provided in the xls file. We will leave it up to the editor's discretion to decide if it should be published or not.

R2 Q2-2.The authors need to verify the fidelity of the luciferase reporter by demonstrating that luciferase expression tracks with Dicer expression in a system where differential Dicer expression is already established. Similarly, the authors need to include a luciferase construct for a different (non-Dicer) promoter and demonstrate it does not respond to UV/bcat, etc. These are basic controls.

R2 A2-2) This luciferase reporter construct was published in a *Cell* paper (Levy et al., 2010). Since it was published in a very high-profile journal, all the appropriate basic controls were performed. In this paper, the authors clearly demonstrated that the expression of Dicer was positively regulated by MITF. They showed that overexpression of MITF in UACC62 melanoma cells resulted in increased Dicer::luciferase activity, whereas MITF knockdown decreased Dicer::luciferase activity. Our experiments testing the effect of UV on the TOP-flash reporter were done using the FOP-flash mutant construct as a negative and normalization control. This FOP-flash reporter construct was non-responsive to UV. By definition *a priori*, it is not a trivial or simple task to choose a reporter construct that does not respond to UV, as this will depend on the experimental system.

© 2024. Published by The Company of Biologists under the terms of the Creative Commons Attribution License (https://creativecommons.org/licenses/by/4.0/).

R2 Q2-3.GAPDH is a curious choice of a housekeeping gene. It is acknowledged that it is frequently used for this purpose, but regardless, it is prudent to consider the system you are using. In this case, the expression of GAPDH is regulated by both Pi3K signaling and p53 (reviewed in PMID: 25859407). Might the reduction in relative Dicer be the consequence of an increase in GAPDH? For all RT-qPCR data:

R2 A2-3) As mentioned above, we have used two reference genes for the mouse cell lines, and three for the human cell lines. The review which the referee cited describes how activation of PI3K affects the phosphorylation of GAPDH protein, but no mention is made of *GAPDH* mRNA expression being regulated by PI3K. Furthermore, the review references that GAPDH enzymatic activity can be altered by UV irradiation, but does not describe the effect on *GAPDH* expression.

Nonetheless, as shown in the attached xls file with the raw data, the levels of GAPDH following UV do not change. As such, the observed effect of UV on Dicer is always consistent, and not a result of alterations in the levels of housekeeping genes.

R2 Q2-3a. Try a second housekeeping gene that is not regulated by the pathways being queried.

R2 A2-3a) See above, we used *GAPDH*, *SDHA* and *TBP* for human cell lines, and *Gapdh* and *Hprt* for mouse cell lines.

R2 Q2-3b. Try a second experimental gene besides Dicer, this is not regulated by the pathways being queried to determine whether its expression appears stable when made relative to GAPDH

R2 A2-3b) Here, we followed Dicer, Gapdh, Sdha, Hprt and Tbp, and luciferase constructs including Dicer::luciferase, Top-flash and Fop-flash. Overall, the presented results are consistent with the regulation of Dicer by UVB. Even though, when there is a decrease of the controls, the decrease of Dicer is more dramatic. At this point, we truly trust our general conclusions.

R2 Q2-3c.Provide the raw data - including representative amplification and melting curves as well as the Ct values for each experiment, so that it can be assessed by the reader. These are just the minimal requirements of the MIQE guidelines (PMID: 19246619)

R2 A2-3c) We have provided the raw Ct values for all qPCR experiments in the xls file. Below, we are providing one representative PCR amplification plot and melting curves for a qPCR assessment of Dicer expression in MNT-1 cells. We respectfully note that providing amplification and melting curves are not a requirement of *JCS* and are not required by the vast majority of journals.

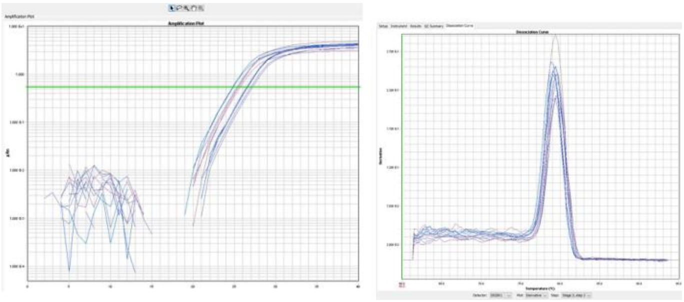

Left panel: Amplification plot that displays the Log (DRn) graphed versus cycle number. The green line represents the threshold. Right panel: Dissociation curve plot that displays the Derivative reporter (change in fluorescence/change in temperature) graphed versus temperature. Note the presence of a single peak for the amplified products.

R2 Q2-3d.A consistent housekeeping loading control would strengthen the manuscript - as is, it is not clear what loading control is used for which experiment (GAPDH, TBP, Actin, etc), but whatever loading controls the authors use, it is necessary to show that their expression, both RNA and protein, are not affected by UV dosing - similar to Fig. 1A and B.

© 2024. Published by The Company of Biologists under the terms of the Creative Commons Attribution License (https://creativecommons.org/licenses/by/4.0/).

R2 A2-3d) The referee is right, we adapted our test according to the cellular models that we used. Indeed, TBP is sensitive to UV in 501Mel cell line, which was not the case for GAPDH nor SDHA. In Figure 1, all RT-qPCR data is normalized to Gapdh. In Figure S1, we used various normalization controls to demonstrate that the decrease of Dicer following UVB exposure is real even though the normalization control may move. The essential is that even when using three different normalization controls, we observe that UVB decreases Dicer expression. Below we are showing the Cts for 501Mel in the presence or absence of UVB using TBP (left panel), SDHA (center panel), and GAPDH (right panel).

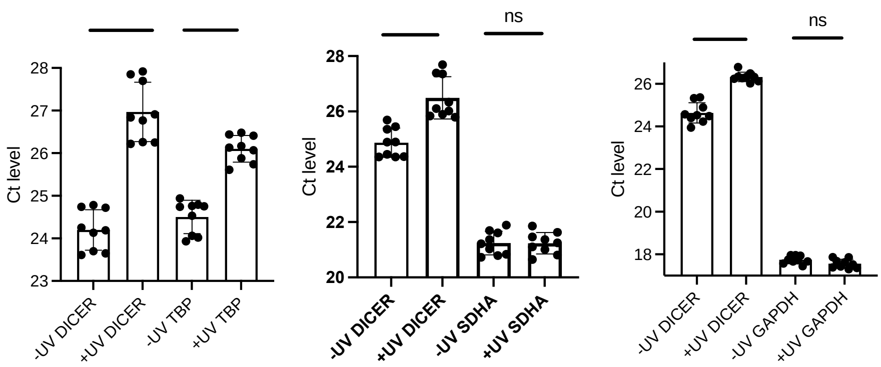

The primary objective is to demonstrate a relative alteration in the level of Dicer compared to a reference. Here, we illustrate that SDHA and GAPDH exhibit no RNA-level modifications, and the level of TBP is relatively less affected than that of Dicer. When the gene of interest is more affected than the reference gene, it indicates a genuine impact on the gene of interest under these conditions.

Regarding the Western blot analysis, we quantified the protein amounts using the BCA assay before loading approximately equal amounts of proteins into each well. Vinculin was used as a loading control for Dicer western blots.

R2 Q2-3e. To be clear – much of this manuscript hinges on the interpretation of RT-qPCR data. In almost all panels, the RT-qPCR data presented show a 2x change in signal, which is the equivalent of a single cycle / Ct. In some cases (Fig 2H) the authors claim p<0.001 significance between a relative RT-qPCR value of 1 compared to 1.3 – which is less than one Ct difference and thereby less than the theoretical sensitivity of the assay. The authors, therefore, are required to provide transparency and quality control of their RT-qPCR to present a convincing argument. I will reiterate that transparency of both the assay/data as well as the statistics (as discussed in point 1) are required.

R2 A2-3e) We believe that we have addressed these issues.

R2 Q2-4.Figure 1D, as is, does not support the interpretation that 'the level of Dicer protein was decreased across transformed and non-transformed cell lines.' In particular, the melan-a decrease is non-obvious and the associated actin bands do not appear to match. The methods state that western blots were all conducted at least three times. It is necessary to provide these data in the form of both raw scans and semi-quantification.

R2 A2-4) The referee is right. We have now quantified our western blot data and added the statistics. As mentioned by the second reviewer, the western blots were not profoundly convincing for an external eye. We therefore acted on this issue; besides changing the batch of antibodies, we improved the protocols to optimize the relative differences of expression of Dicer and observed that 27 hours after irradiation, decreases in the levels of Dicer were more apparent. The reduction of the level of Dicer protein is around 50%. The different western blots are shown and presented as Western blot transparency.

R2 Q2-5.Related to Figs 2 and 3: The authors need to demonstrate that activation of P13K, RSK and GSK3B also increase Dicer expression and promoter activity, as would be expected if their hypothesis were true.

© 2024. Published by The Company of Biologists under the terms of the Creative Commons Attribution License (https://creativecommons.org/licenses/by/4.0/).

R2 A2-5) Since this is a short report, we did not have the intention to show and dissect all of the pathways involved in the regulation of Dicer expression. We demonstrated that these pathways can affect Dicer expression, but what is essential is that these pathways converge on ß-catenin. Our hypothesis was that ß-catenin is implicated in regulating Dicer expression. We tested this in Figure 2F-H, demonstrating that ß-catenin plays a role in decreasing Dicer expression downstream of UV irradiation. The question of the referee, even of importance, remains out of the scope of this article.

However, to complement our experiments in Figure 2A-C, we used inhibitors of the three pathways involved in regulating Dicer expression (PI3K, RSK and GSK3ß) and assessed the effects on Dicer::luciferase activity. Our results are in agreement with the mRNA data, which show that the inhibition of these pathways decreases Dicer expression. We added these results in the new Figure 2D and Figure S2D.

R2 Q2-6. The authors should measure the pre-miRNA and mature miRNA for well-expressed miRNAs in the +/- UV and pathway inhibitor and activating conditions. If their hypothesis were accurate, an increase in pre and a decrease in mature would be expected and would verify the presented results.

R2 A2-6) We did not follow the pre-miRNA and mature miRNAs since, being a short report, our study was limited to Dicer. At this point, we are wondering if this *Short report* should not be a full article.

We respectfully disagree with the referee regarding the fact that we should have an increase in the pre-miRNA. At this point, it is not clear what effect UVB treatment would have on the expression of the pre-miRs. We do expect that UVB would decrease the levels of mature miRNAs, as it decreases Dicer levels. It is indeed possible that following UVB, there would be an increase in the pre-miR levels, if there was a molecular compensation for the decreased levels of mature miRNAs, but it may not be.

We chose to look at the expression of three miRNAs, (miR92b, miR107, miR188), which previously have been shown to be positively regulated by Dicer in melanocytes (Levy et al., 2010; Bertrand et al., 2024). We hypothesized that UVB, which decreases Dicer expression, would decrease the levels of these miRNAs, in comparison with the associated pre-miRNA gene expression. Indeed, we observed that in MNT1 cells, UVB decreased the expression of the mature miR92b to 0.13 compared to non-irradiated controls (Fig. 2I), whereas the expression of the pre-miR92b was decreased only to 0.55. Similarly, expression of the mature miR107 was decreased to 0.22 following UVB, whereas UVB decreased pre-miR107 expression only to 0.52. In these two cases, while UVB decreased the expression of the pre-miR, the subsequent decrease in the mature miR expression was to a higher magnitude. UVB also decreased the expression of miR188 to 0.28, whereas the expression of pre-miR188 significantly increased (1.92) compared to non-irradiated controls (Fig. 2I).

Minor:
R2 Q2-7.The text indicates that Dicer mRNA was reduced after UV exposure in WM852 cells, citing Fig. 1C, but there are no data to support this claim.

R2 A2-7) We thank the referee for this point. We have removed WM852 cells from this article. Since, we fully modified our western blot protocol and readdressed the question, we used another human melanoma cell line, SK28. All requested information is included.

R2 Q2-8.Figure 1 “F”, should be Fig 1 “E”

R2 A2-8) Done.

Second decision letter

© 2024. Published by The Company of Biologists under the terms of the Creative Commons Attribution License (https://creativecommons.org/licenses/by/4.0/).

MS ID#: JOCES/2024/261978

MS TITLE: UVB radiation suppresses Dicer expression through β-catenin

AUTHORS: Zackie Aktary, Valerie Petit, Irina Berlin, Nisa Charenchaon, Evelyne Sage, Juliette U. Bertrand, and Lionel Larue

We have now reached a decision on the above manuscript.

To see the reviewers' reports and a copy of this decision letter, please go to: https://submit-jcs.biologists.org and click on the 'Manuscripts with Decisions' queue in the Author Area. (Corresponding author only has access to reviews.)

As you will see, one of the referees is satisfied with the revised manuscript, but the other referee still has some substantive concerns that I trust you will be able to address.

The major issue I would highlight relates to the statistical analysis. I refer you to the point made by the referee that equalizing the control values to 1 could artificially reduce the variance, violating the assumption of the t-test you used. This holds true for all the data analyzed in this way, not just the experiment in question (as the referee states).

My recommendation to you would be to consult with a statistical expert at your institution, to either provide guidance in re-analyzing the data or to provide an explanation of how the data were analyzed (if you think the referee misunderstood how the statistical analysis was done). Of the reviewers' comments, this is my primary concern, although I would advise you address the other issues as far as possible.

Please ensure that you clearly highlight all changes made in the revised manuscript. Please avoid using 'Tracked changes' in Word files as these are lost in PDF conversion.

I should be grateful if you would also provide a point-by-point response detailing how you have dealt with the points raised by the reviewers in the 'Response to Reviewers' box. Please attend to all of the reviewers' comments. If you do not agree with any of their criticisms or suggestions please explain clearly why this is so.

Reviewer 1

Advance summary and potential significance to field

In their manuscript, Akatary et al studied the regulation of DICER by UVB radiation. They demonstrate that UVB inhibits DICER expression in various cell types. This effect is transcriptional and mediated by the LEF/β-Catenin complex.
Given the central role of DICER in cell biology, and its regulation by numerous stresses, it is of paramount importance to describe the regulation of DICER by UVB. Indeed, UVB is one of the most harmful compulsory environmental stimuli responsible for a wide range of skin diseases, including cancer and premature skin aging conditions.
The revised version takes into account all my comments and requests. The paper has been globally clearly improved and is now acceptable for publication in the Journal of Cell Science.

Comments for the author

Thank you for taking into account my comments and for having improved the paper quality.

Reviewer 2

© 2024. Published by The Company of Biologists under the terms of the Creative Commons Attribution License (https://creativecommons.org/licenses/by/4.0/).

Advance summary and potential significance to field

The authors have made significant progress in addressing the majority of my concerns, including the addition of data that reinforces their central hypothesis. The experiments presented are well-conceived and generally support the key conclusions of the study. In this regard, I believe this report has the potential to be a valuable contribution to the field.

Comments for the author

However, I still have some reservations about the integrity of several conclusions drawn in the manuscript. While I understand the authors' frustration, I must express, with all due respect, that there appear to be some issues with the transparency and completeness of the data presented. These concerns stem primarily from the application of inappropriate statistical tests and the absence of certain control and raw data, which the authors have chosen not to include.

I discuss the statistics more below. Regarding control/raw data, the authors have justified their decisions to omit with arguments such (paraphrased here) as “controls weren’t required because the plasmid was published in a high-profile journal,” “raw data are not required because journals don’t usually ask for this,” and “validation is not required because this is a Short Report.” While I empathize with the authors’ position, these justifications are increasingly at odds with current editorial standards.

To be clear, I fully recognize that historically, many journals have not mandated raw data, have permitted the use of less rigorous statistical methods, and have accepted manuscripts without exhaustive controls. However, we are in a period of heightened scrutiny, where retraction rates are rising, and major journals are becoming more stringent about the application of proper statistical analyses and the transparency of data.

In summary, while I acknowledge that the arguments presented by the authors may have been acceptable in the past and might still be accepted in some venues, the current editorial climate demands greater rigor.
The authors’ reluctance to provide raw data and to apply appropriate statistical methods, based on the premise that these requirements are atypical or only necessary for a Full Article, is ultimately at the editor’s discretion.

Major concern:

“R2 A2-1 f) We had performed our statistical analyses using the normalized data.
However, we do understand the concern of the referee. For that reason, we reanalyzed our data using the unpaired t-test with Welch’s correction. This correction accommodates for unequal variance between groups. Overall, this correction changed the p-value of our results, but it did not change the significance of the results. We have to moderate this conclusion for one case: Figure 1B when we tested the dose increment between 10 and 25 mJ/cm2, since the unpaired t-test p-value was close to 0.05, the Welch’s test indicated that the results were not significant.

For information, experiments were conducted using at least three independent biological samples, each with two/three technical replicates. Naturally, the quantities of mRNA and resultant cDNA can fluctuate between biological experiments, potentially leading to artificial differences devoid of biological significance. For this reason, we chose to show normalized values.”

I’m afraid this approach is still not statistically justified. When you normalize data in such a way that all control values are equalized to 1, you're essentially stripping away the natural variability that exists between your experimental conditions. This artificial reduction of variance can lead to misleading statistical conclusions. As the authors state, Welch's t-test is designed to handle cases where there is unequal variance between groups, but it still assumes that the data points within each group are independent and that the variance within groups is reflective of the true variance in the population. If you normalize the data to remove all variance, this assumption is violated because the true variability of the data is no longer represented. When performing statistical tests especially those based on t-tests, it's important to preserve the natural variance in the data. If

© 2024. Published by The Company of Biologists under the terms of the Creative Commons Attribution License (https://creativecommons.org/licenses/by/4.0/).

normalization is necessary, it should be done in a way that does not completely eliminate this variance (e.g., using a z-score normalization that preserves relative differences). Transformations that stabilize variance (like log or square root transformations) are sometimes justified if the data has a skewed distribution, but again they don't eliminate variance—they just make it more consistent across groups.

The justification presented by the authors for using the normalized values that eliminate variance is that: “three independent biological samples, each with two/three technical replicates. Naturally, the quantities of mRNA and resultant cDNA can fluctuate between biological experiments.” To be clear, this is true of every single properly conducted biological experiment ever. That independent experiments were conducted, and biological noise was observed is just what happens when running an experiment, it is not a reason to conduct improper statistics. I recommend you use the triplicate data you have to conduct a proper power calculation to then determine how many more biological replicates you need to run to have confidence in a 2-fold affect size.

To be clear – this is not just in reference to qPCR data. Literally every plot in this manuscript shows a normalization of controls to 1 (and by in large effect sizes of 2 or less).
Again, this is fine for visualization, but inappropriate if these normalize values were used for the statistics. Can the authors verify the appropriate use of statistical tests (as described above) for all comparisons?

To summarize – the statistical test strategy here used is incorrect. That isn’t up for debate, it’s just fact. However, as the authors have stated, this approach has been ‘allowed’ in the past (and probably present) by many journals, and this is a “Short Report” as the authors have argued. I believe this falls more into the category of editorial policy than a recommendation I can make. Stats aren’t technically correct, but they are ‘correct enough’ to often pass review – if that’s okay by policy by today’s standards, then I would recommend for publication. If not, then I would not recommend for publication and would be happy to rereview, but only if the authors make a good faith effort to conduct the statistics correctly.

These following are minor issues that do not necessarily need to be addressed but we encourage the authors to consider it to further demonstrate the rigor of their results:

R2 A2-3c) We have provided the raw Ct values for all qPCR experiments in the xls file. Below, we are providing one representative PCR amplification plot and melting curves for a qPCR assessment of Dicer expression in MNT-1 cells. We respectfully note that providing amplification and melting curves are not a requirement of JCS and are not required by the vast majority of journals.

Left panel: Amplification plot that displays the Log (DRn) graphed versus cycle number. The green line represents the threshold. Right panel: Dissociation curve plot that displays the Derivative reporter (change in fluorescence/change in temperature) graphed versus temperature. Note the presence of a single peak for the amplified products.

It appears that there may have been a file conversion error on the web portal’s end, as I am unable to see the plots referenced in the manuscript. Nevertheless, I find it difficult to understand the authors' reluctance to provide the raw plots as supplementary data. While it is true that such data has not been historically required (as noted in my earlier comments), the authors are using this assay to draw conclusions based on very small changes in RNA expression. Including a representative disassociate plot for each probe set would be a valuable addition.

Moreover, I am also puzzled by the hesitation to include the amplification curves. These curves would only serve to strengthen the presented data and hypothesis, thereby addressing the concerns of critical reviewers like myself. Providing this additional information could only enhance the manuscript’s credibility and robustness, so I encourage the authors to consider including it.
That being said it is as the authors argue, an unusual request – so discretion of the editor.

Minor example
R2 A2-2) This luciferase reporter construct was published in a Cell paper (Levy et al., 2010). Since it was published in a very high-profile journal, all the appropriate basic controls were performed. In

© 2024. Published by The Company of Biologists under the terms of the Creative Commons Attribution License (https://creativecommons.org/licenses/by/4.0/).

this paper, the authors clearly demonstrated that the expression of Dicer was positively regulated by MITF.
They showed that overexpression of MITF in UACC62 melanoma cells resulted in increased Dicer::luciferase activity, whereas MITF knockdown decreased Dicer::luciferase activity. Our experiments testing the effect of UV on the TOP-flash reporter were done using the FOP-flash mutant construct as a negative and normalization control. This FOP-flash reporter construct was non-responsive to UV. By definition a priori, it is not a trivial or simple task to choose a reporter construct that does not respond to UV, as this will depend on the experimental system.

I find the response regarding the plasmid's origin from a 'high profile' publication difficult to reconcile. The use of a published plasmid does not eliminate the need for standard controls. While I acknowledge that it may not be trivial to find reporter constructs that do not respond to UV, this challenge only underscores the importance of including appropriate controls in a study on the cellular response to UV.

That said, given the increased robustness of the other data provided, the fact that the luciferase assay remains less than robust is not as significant. The conclusions of the study can still be effectively communicated without relying on this particular assay.

Second revision

Author response to reviewers' comments

Reviewer 1 Advance summary and potential significance to field

In their manuscript, Akatary et al studied the regulation of DICER by UVB radiation. They demonstrate that UVB inhibits DICER expression in various cell types. This effect is transcriptional and mediated by the LEF/β-Catenin complex.
Given the central role of DICER in cell biology, and its regulation by numerous stresses, it is of paramount importance to describe the regulation of DICER by UVB. Indeed, UVB is one of the most harmful compulsory environmental stimuli, responsible for a wide range of skin diseases, including cancer and premature skin aging conditions.
The revised version takes into account all my comments and requests. The paper has been globally clearly improved and is now acceptable for publication in the Journal of Cell Science.

Reviewer 1 Comments for the author
Thank you for taking into account my comments and for having improved the paper quality.

Answer: We thank the reviewer for the positive comments.

Reviewer 2 Advance summary and potential significance to field
The authors have made significant progress in addressing the majority of my concerns, including the addition of data that reinforces their central hypothesis. The experiments presented are well-conceived and generally support the key conclusions of the study. In this regard, I believe this report has the potential to be a valuable contribution to the field.

Answer: Thank you to the reviewer for their thoughtful and encouraging comments. We are pleased to hear that the additional data and experiments have effectively addressed the majority of the concerns and that our key conclusions are supported. We appreciate the reviewer's recognition of the potential value of our work to the field.

Reviewer 2 Comments for the author
However, I still have some reservations about the integrity of several conclusions drawn in the manuscript. While I understand the authors' frustration, I must express, with all due respect, that there appear to be some issues with the transparency and completeness of the data presented. These concerns stem primarily from the application of inappropriate statistical tests and the absence of certain control and raw data, which the authors have chosen not to include. I discuss

© 2024. Published by The Company of Biologists under the terms of the Creative Commons Attribution License (https://creativecommons.org/licenses/by/4.0/).

the statistics more below. Regarding control/raw data, the authors have justified their decisions to omit with arguments such (paraphrased here) as “controls weren’t required because the plasmid was published in a high-profile journal,” “raw data are not required because journals don’t usually ask for this,” and “validation is not required because this is a Short Report.” While I empathize with the authors’ position, these justifications are increasingly at odds with current editorial standards.

To be clear, I fully recognize that historically, many journals have not mandated raw data, have permitted the use of less rigorous statistical methods, and have accepted manuscripts without exhaustive controls. However, we are in a period of heightened scrutiny, where retraction rates are rising, and major journals are becoming more stringent about the application of proper statistical analyses and the transparency of data.

In summary, while I acknowledge that the arguments presented by the authors may have been acceptable in the past and might still be accepted in some venues, the current editorial climate demands greater rigor. The authors’ reluctance to provide raw data and to apply appropriate statistical methods, based on the premise that these requirements are atypical or only necessary for a Full Article, is ultimately at the editor’s discretion.

Major concern:
“R2 A2-1 f) We had performed our statistical analyses using the normalized data. However, we do understand the concern of the referee. For that reason, we reanalyzed our data using the unpaired t-test with Welch’s correction. This correction accommodates for unequal variance between groups. Overall, this correction changed the p-value of our results, but it did not change the significance of the results. We have to moderate this conclusion for one case: Figure 1B when we tested the dose increment between 10 and 25 mJ/cm2, since the unpaired t-test p-value was close to 0.05, the Welch’s test indicated that the results were not significant.
For information, experiments were conducted using at least three independent biological samples, each with two/three technical replicates. Naturally, the quantities of mRNA and resultant cDNA can fluctuate between biological experiments, potentially leading to artificial differences devoid of biological significance. For this reason, we chose to show normalized values.”

I’m afraid this approach is still not statistically justified. When you normalize data in such a way that all control values are equalized to 1, you're essentially stripping away the natural variability that exists between your experimental conditions. This artificial reduction of variance can lead to misleading statistical conclusions. As the authors state, Welch's t-test is designed to handle cases where there is unequal variance between groups, but it still assumes that the data points within each group are independent and that the variance within groups is reflective of the true variance in the population. If you normalize the data to remove all variance, this assumption is violated because the true variability of the data is no longer represented. When performing statistical tests, especially those based on ttests, it's important to preserve the natural variance in the data. If normalization is necessary, it should be done in a way that does not completely eliminate this variance (e.g., using a z-score normalization that preserves relative differences). Transformations that stabilize variance (like log or square root transformations) are sometimes justified if the data has a skewed distribution, but again, they don't eliminate variance—they just make it more consistent across groups. The justification presented by the authors for using the normalized values that eliminate variance is that: “three independent biological samples, each with two/three technical replicates. Naturally, the quantities of mRNA and resultant cDNA can fluctuate between biological experiments.” To be clear, this is true of every single properly conducted biological experiment ever. That independent experiments were conducted, and biological noise was observed is just what happens when running an experiment, it is not a reason to conduct improper statistics. I recommend you use the triplicate data you have to conduct a proper power calculation to then determine how many more biological replicates you need to run to have confidence in a 2-fold affect size.
To be clear – this is not just in reference to qPCR data. Literally every plot in this manuscript shows a normalization of controls to 1 (and by in large effect sizes of 2 or less). Again, this is fine for visualization, but inappropriate if these normalize values were used for the statistics. Can the authors verify the appropriate use of statistical tests (as described above) for all comparisons?

© 2024. Published by The Company of Biologists under the terms of the Creative Commons Attribution License (https://creativecommons.org/licenses/by/4.0/).

To summarize – the statistical test strategy here used is incorrect. That isn't up for debate, it's just fact. However, as the authors have stated, this approach has been 'allowed' in the past (and probably present) by many journals, and this is a "Short Report" as the authors have argued. I believe this falls more into the category of editorial policy than a recommendation I can make. Stats aren't technically correct, but they are 'correct enough' to often pass review – if that's okay by policy by today's standards, then I would recommend for publication. If not, then I would not recommend for publication, and would be happy to rereview, but only if the authors make a good faith effort to conduct the statistics correctly.

Answer: We fully agree with the reviewer's important point regarding the current emphasis on heightened scrutiny and the rising retraction rates in scientific publishing. We understand that the key concern is the validity of our statistical analyses. We feel there is clear misunderstanding between the referee and us. The reviewer mentions that we are "reluctant to provide raw data". In our previous submission, we provided comprehensive and transparent information by giving all xls files with the main data. We now included all raw data as Table S2. If the reviewer had problem to open these files, it would have been fair to ask directly the editor since this short report was sent on mid-July.

However, we recognize that the reviewer still has concerns about our approach. We consulted with statisticians in Curie and abroad and used the Wilcoxon test from the raw data that are now included as supplemental tables. We included the statisticians as authors in the article.

Minor issues

These following are minor issues that do not necessarily need to be addressed, but we encourage the authors to consider it to further demonstrate the rigor of their results:

R2 A2-3c) We have provided the raw Ct values for all qPCR experiments in the xls file. Below, we are providing one representative PCR amplification plot and melting curves for a qPCR assessment of Dicer expression in MNT-1 cells. We respectfully note that providing amplification and melting curves are not a requirement of JCS and are not required by the vast majority of journals. Left panel: Amplification plot that displays the Log (DRn) graphed versus cycle number. The green line represents the threshold. Right panel: Dissociation curve plot that displays the Derivative reporter (change in fluorescence/change in temperature) graphed versus temperature. Note the presence of a single peak for the amplified products. It appears that there may have been a file conversion error on the web portal's end, as I am unable to see the plots referenced in the manuscript. Nevertheless, I find it difficult to understand the authors' reluctance to provide the raw plots as supplementary data. While it is true that such data has not been historically required (as noted in my earlier comments), the authors are using this assay to draw conclusions based on very small changes in RNA expression. Including a representative disassociate plot for each probe set would be a valuable addition.
Moreover, I am also puzzled by the hesitation to include the amplification curves. These curves would only serve to strengthen the presented data and hypothesis, thereby addressing the concerns of critical reviewers like myself. Providing this additional information could only enhance the manuscript's credibility and robustness, so I encourage the authors to consider including it. That being said it is, as the authors argue, an unusual request – so discretion of the editor.

Answer: We have now included representative amplification plots and melting curves for each primer sets used as supplemental S5.

Minor example R2 A2-2) This luciferase reporter construct was published in a Cell paper (Levy et al., 2010). Since it was published in a very high-profile journal, all the appropriate basic controls were performed. In this paper, the authors clearly demonstrated that the expression of Dicer was positively regulated by MITF. They showed that overexpression of MITF in UACC62 melanoma cells resulted in increased Dicer::luciferase activity, whereas MITF knockdown decreased Dicer::luciferase activity. Our experiments testing the effect of UV on the TOP-flash reporter were done using the FOP-flash mutant construct as a negative and normalization control. This FOP-flash reporter construct was non-responsive to UV. By definition a priori, it is not a trivial or simple task to choose a reporter construct that does not respond to UV, as this will depend on the experimental system.

© 2024. Published by The Company of Biologists under the terms of the Creative Commons Attribution License (https://creativecommons.org/licenses/by/4.0/).

I find the response regarding the plasmid's origin from a 'high profile' publication difficult to reconcile. The use of a published plasmid does not eliminate the need for standard controls. While I acknowledge that it may not be trivial to find reporter constructs that do not respond to UV, this challenge only underscores the importance of including appropriate controls in a study on the cellular response to UV. That said, given the increased robustness of the other data provided, the fact that the luciferase assay remains less than robust is not as significant. The conclusions of the study can still be effectively communicated without relying on this particular assay.

Answer: We appreciate that the reviewer recognized that this is a minor issue and as mentioned by the referee the conclusions of the study can still be effectively communicated.

Third decision letter

MS ID#: JOCES/2024/261978

MS TITLE: UVB radiation suppresses Dicer expression through β-catenin

AUTHORS: Zackie Aktary, Valerie Petit, Irina Berlin, Jeremy H. Raymond, Frederique Berger, Nisa Charenchaon, Evelyne Sage, Juliette U. Bertrand, and Lionel Larue

I am happy to tell you that your manuscript has been accepted for publication in Journal of Cell Science, pending standard publication integrity checks.

© 2024. Published by The Company of Biologists under the terms of the Creative Commons Attribution License (https://creativecommons.org/licenses/by/4.0/).
